# Delta-band audience brain synchrony tracks engagement with live and recorded dance

**DOI:** 10.1016/j.isci.2025.112922

**Published:** 2025-07-07

**Authors:** Laura A. Rai, Haeeun Lee, Emma Becke, Carlos Trenado, Sonia Abad-Hernando, Matthias Sperling, Diego Vidaurre, Melanie Wald-Fuhrmann, Daniel C. Richardson, Jamie A. Ward, Guido Orgs

**Affiliations:** 1 Institute of Cognitive Neuroscience, University College London, Alexandra House, 17 Queen Square, London, UK; 2 Department of Psychology, Goldsmiths, University of London, Lewisham Way, New Cross, London, UK; 3 Department of Music, Max Planck Institute for Empirical Aesthetics, Frankfurt am Main, Germany; 4 Independent Artist & Choreographer, London, UK; 5 Siobhan Davies Studios, 85 St George’s Road, SE1 6ER London, UK; 6 Department of Clinical Medicine - Center of Functionally Integrative Neuroscience, Aarhus University, Aarhus, Denmark; 7 Department of Psychiatry, University of Oxford, UK; 8 Department of Experimental Psychology, University College London, London, UK; 9 Department of Computing, Goldsmiths, University of London, Lewisham Way, New Cross, London, UK

**Keywords:** Natural sciences, Biological sciences, Neuroscience, Behavioral neuroscience, Clinical neuroscience, Cognitive neuroscience

## Abstract

Evolutionary theories claim that dance and music have evolved as collective rituals for social bonding and signaling. Yet, neuroscientific studies of these art forms typically involve people watching video or sound recordings alone in a laboratory. Across three live performances of a dance choreography, we simultaneously measured real-time dynamics between the brains of up to 23 audience members using mobile wet-electrode EEG. Interpersonal neural synchrony (INS) in the delta band (1–4 Hz) was highest when performers directly interacted with audience members (breaking the fourth wall) and varied systematically with the dancers’ movements and artistically predicted and actual continuous engagement. In follow-up studies using video recordings of the performance, we show that audience brain synchrony and engagement are highest when dance is experienced live and together. Our study shows that the ancient social functions of the performing arts are preserved in engagement with contemporary dance.

## Highlights

• EEG Delta-band inter-brain synchrony tracks engagement with live/recorded dance
• Dance artists can predict audience engagement and brain synchrony over time
• Audience brain synchrony is highest when dancers directly interact with spectators
• Alpha/Delta EEG power is reduced if people watch dance together rather than alone

## Graphical abstract

**Figure:**
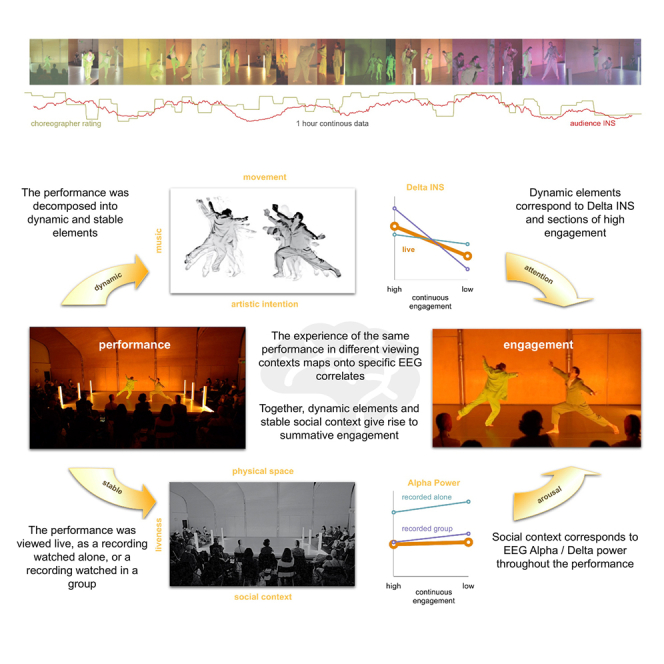

## Introduction

Across the world, live performances of theater, dance and music attract large and growing numbers of visitors.^1^^,^^2^^,^^3^ The phenomenon and experience of live events have been discussed from both theoretical^4^^,^^5^^,^^6^ and empirical perspectives,^7^^,^^8^ yet a cognitive neuroscience approach to understanding the appeal of live events is difficult, as traditional data collection and analysis methods are based on the repetition of multiple short experimental trials in a laboratory context. Two specific features of live events are particularly challenging from a neuroscience perspective. Firstly, live performances are *physically live* in that they are unique, non-reproducible, and can only be experienced once in the exact same way.^9^^,^^10^^,^^11^ Secondly, performances are *socially live* in that they are typically experienced in groups.^5^^,^^12^^,^^13^ Neuroimaging studies have only recently started to focus on live social interactions, but with a few exceptions are typically limited to two people,^14^ rather than the large groups that attend a live event.

Dance and music have evolved as participatory performances^12^^,^^15^^,^^16^^,^^17^^,^^18^^,^^19^ and rituals^20^ that communicate or facilitate group affiliation.^21^^,^^22^^,^^23^ Contemporary dance makers often explicitly reference the spiritual and ritualistic origins of dance by foregrounding altered states of consciousness, non-linear narrative, participation, improvisation, or immersion as core aesthetic and creative features.^10^^,^^24^^,^^25^^,^^26^ Dance communicates nonverbal information through movement,^13^^,^^27^ and evokes synchronized brain activations in individual spectators. Social interactions might also drive higher engagement of audiences in live^28^^,^^29^^,^^30^^,^^31^ or streamed^32^ music concerts, but it is unknown to what extent communication of performance content (movement, music, or artistic intentions) depends on performance context (physical and social liveness).

Existing neurocognitive research on engagement with temporal art forms (dance, music or film) typically involves measuring continuous brain activity from individuals watching video or sound recordings alone in a lab.^33^^,^^34^^,^^35^^,^^36^^,^^37^^,^^38^^,^^39^ Synchrony of brain activity between individuals across time has been proposed as an index of shared attentional engagement while watching emotionally arousing or engaging video clips or film scenes with both EEG^40^ and fMRI.^39^^,^^41^^,^^42^^,^^43^ Indeed, greater EEG brain synchrony predicts superior memory recall and is diminished by distraction from external tasks.^44^^,^^45^ From a joint attention perspective on engagement, brain synchrony reflects neural processing similarities of a shared stimulus, rather than a genuinely shared affective experience.^46^ Dikker et al.^47^ show that students’ brain synchrony, measured as alpha coherence, relative to others in a classroom, increases with the students’ individual arousal. In this study, we deploy the joint attention account of engagement to better understand the role of liveness for the experience of dance. We commissioned a dance performance titled *Detective Work,* created by choreographer Seke Chimutengwende in collaboration with dance artist Stephanie McMann, which incorporates elements of theater and music as well as moments of direct interaction with the audience.^48^ Across three live performances, we collected simultaneous mobile EEG recordings from up to 23 audience members (Final *N* = 59) and measured their interbrain synchrony in relation to (a) continuous objective features of the performance: dancer acceleration and distance, lighting and soundtrack (b) subjective ratings of audience engagement as predicted by the choreographer, performer, and the dramaturg and (c) the audience’s breathing synchrony. More detailed information on the artistic process of making *Detective Work* is available from https://neurolive.info/Performance-1. To assess the role of both physical and social liveness, we also conducted a series of follow-up studies where people watched a video recording of the dance performance on their own in a laboratory setting, or together as a group in the dance studio or a cinema.

We addressed three main research questions. Firstly, by collecting mobile EEG from large groups during live and screened dance performances, we tested whether audience brain synchrony can capture continuous engagement with the performance. In contrast to previous EEG work in this area, we take a data-driven approach that retains the temporal information of the dance performance and does not make a priori assumptions about what EEG frequency bands are relevant to attentional engagement (i.e., alpha). Secondly, we explored whether audience brain synchrony is temporally coupled to the dynamic features of the live performance and reflects communication between dancers and audience members. Thirdly, we tested whether continuous and summative engagement and brain synchrony during the live performances are higher compared to watching a recording of the same performance alone in the lab or together with others.

## Results

**Figure 1:**
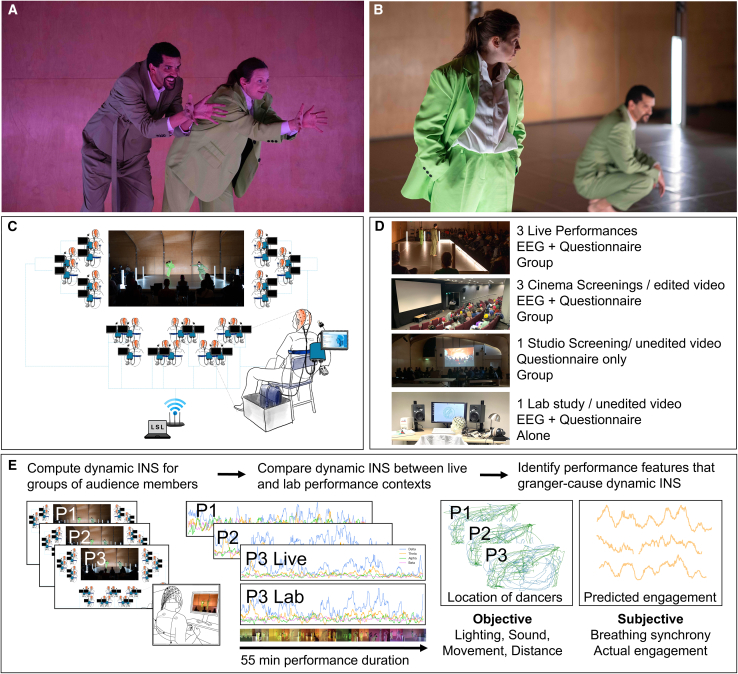
Overview of experimental design and analysis method (A and B) Images of two sections of Detective Work rated as more (A) or less (B) engaging. More images and information are available on www.neurolive.info/Performance-1. Images by Hugo Glendinning. (C) Experimental set-up and stage/seating position of audience members. (D) Datasets: we report EEG and questionnaire data for 3 live performances, a lab study, and questionnaire data for two collective screening studies. (E) Overview of methods and dynamic analysis of interpersonal neural synchrony (INS).

We collected data for three live performances (hereafter P1, P2, P3), three cinema screenings (hereafter S1, S2, S3), a dance studio screening, and a lab study. The sample size for individual cinema screenings did not allow for a group-based time-resolved analysis of brain synchrony; therefore, we only report pairwise analyses of delta phase locking and delta/alpha EEG power for this study. All EEG data for the studio screening were lost due to a data streaming issue (see Figure 1D and Table S1 for an overview of datasets and summary of results). Additionally, we collected predicted and actual continuous engagement ratings for the video of P3 from artists and an independent sample of viewers in an online follow-up study. We report summative engagement collected immediately after the performance for all live and recorded versions of *Detective Work*.

First, we show that summative engagement was highest after watching the dance performances live and together, compared to all screenings and the lab study. Secondly, common sources of EEG signal variation across time and audience members were most pronounced in the EEG delta band for all three live performances. Thirdly, using correlational and granger-causality analyses we relate audience brain synchrony to dynamic performance features and show that inter-brain synchrony systematically varies with both the artists’ predicted and the viewers’ actual continuous engagement. Fourth, we show that delta-band INS was reduced if people watched a video of the performance alone in the lab. Finally, comparing high and low engagement sections of all live performances, the cinema screening, and the lab study, we show that EEG alpha/delta power is lowest and delta phase locking is highest when dance is watched with other people.

### Engagement after the performance is highest when experiencing dance live and together

**Figure 2:**
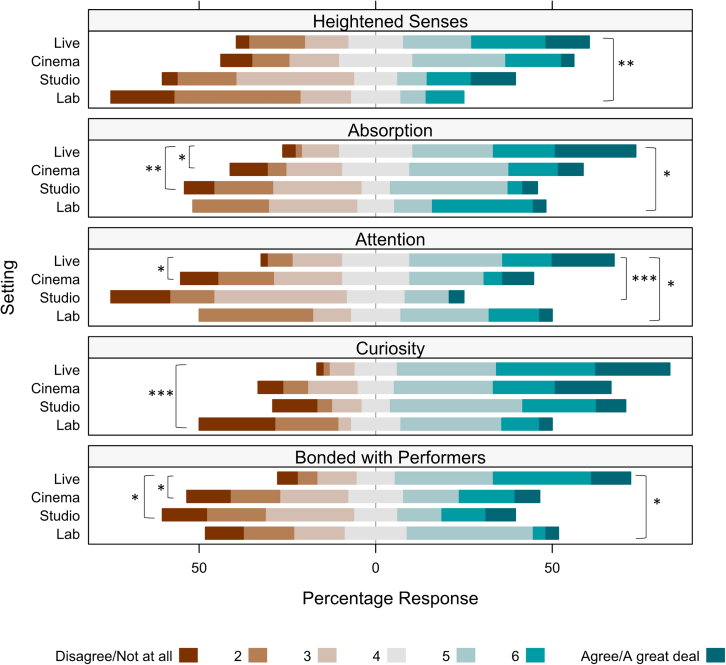
Post-performance questionnaire ratings for audiences in the Live (*N* = 57), lab (*N* = 28), Cinema Screening (*N* = 57), and Studio Screening (*N* = 24) conditions The Studio Screening and Lab experiments presented an unedited version of the video recording, whereas the Cinema Screening presented a professionally edited video. Statistically significant non-parametric comparisons with Mann-Whitney U tests between independent audience groups for the live performance versus two group screenings and the lab study are denoted by ∗∗∗*p_FDR_* < 0.001, ∗∗*p_FDR_* < 0.01, ∗*p*_*FDR*_ < 0.05.

A direct comparison of summative engagement ratings between live performance audience members and viewers in the lab, cinema, and studio screenings reveals that watching live dance together was more engaging than watching recorded versions of *Detective Work* together or alone (see Figure 2). We observed significant differences in responses for “Absorption (‘*I was absorbed in the performance’*)” (*H*(3) = 13.13, *p*_*FDR*_ = 0.03), “Attention (*The performance held my attention*)” (*H*(3) *=* 18.15, *p*_*FDR*_ = 0.01), “Curiosity (*At any moment during the performance, I was curious what would happen next*)” (*H*(3) = 16.73, *p*_*FDR*_ = 0.01), “Heightened Senses (*Attending the performance heightened my senses and made me acknowledge my immediate surroundings more vividly*)” (*H*(3) = 14.72, *p*_*FDR*_ = 0.02), and “Bonded with performers (*To what extent did you relate to, or feel bonded with one or both of the performers?*)” (*H*(3) = 12.04, *p*_*FDR*_ = 0.04). Post-hoc tests showed that watching the live show was associated with experiencing a stronger bond to the performers, greater absorption, and greater attention compared to watching a video recording in the lab (*U =* 1032, *p*_*FDR*_ = 0.01; *U =* 1049, *p*_*FDR*_ = 0.02; *U =* 1023.5, *p*_*FDR*_ = 0.03), the studio screening (*U =* 874.5, *p*_*FDR*_ = 0.02; *U =* 980.5, *p*_*FDR*_ = 0.004; *U =* 1055.5, *p*_*FDR*_ = 0.0004), and the cinema screening (*U =* 355, *p*_*FDR*_ = 0.01; *U =* 383.5, *p*_*FDR*_ = 0.02; *U =* 422, *p*_*FDR*_ = 0.01). Watching the live performances also led to greater awareness of the immediate surroundings and increased curiosity as to what would happen next compared to the lab study (*U =* 1181, *p*_*FDR*_ = 0.001; *U =* 1213.5, *p*_*FDR*_ = 0.0004). Summative engagement immediately after the performance, therefore, depended on both physical (live vs. recorded) and social (watching together or alone) liveness.

In an open-ended question prompting the most memorable moments of the performance, spectators were most likely to remember a performance section in which both dancers directly looked and smiled at individual audience members (“Unison” section, see Figure 1A). This differed significantly between the four performance contexts (χ^2^ = 23.5, *p* < 0.001). More than half of all audience members mentioned the “Unison” section after watching the performance together live (51.47%) with more frequent reporting for the live compared to studio screening (22.22%, χ^2^ = 11.6, *p* < 0.001), and the lab screening (17.86%, χ^2^ = 16.3, *p* < 0.001), but not the cinema screening (43.48%, *p* = 0.41). There was a significantly higher proportion of remembering the Unison section for the lab compared to the cinema screening (χ^2^ = 10.7, *p* = 0.001). These findings suggest a relationship between engagement and memory for specific events of the performance, highlighting the importance of direct social interactions between performers and spectators for audience engagement.

### Live dance performances evoke audience brain synchrony in the EEG delta-band

To identify a neural correlate of continuous audience engagement with live dance, we computed audience brain synchrony using correlated component analysis (CCA, Dmochowski et al.^40^; Parra et al.^45^). Compared to other measures of interpersonal neural synchrony (INS), such as phase lag value, general linear models, coherence, and inter-subject correlations,^49^^,^^50^ CCA provides a group-based and data-driven approach to computing INS that allows to reduce the complexity of our data (1h recordings of 23 participants multiplied by 32-electrode EEG arrays) without making arbitrary assumptions about specific frequency windows or electrode locations.

**Figure 3:**
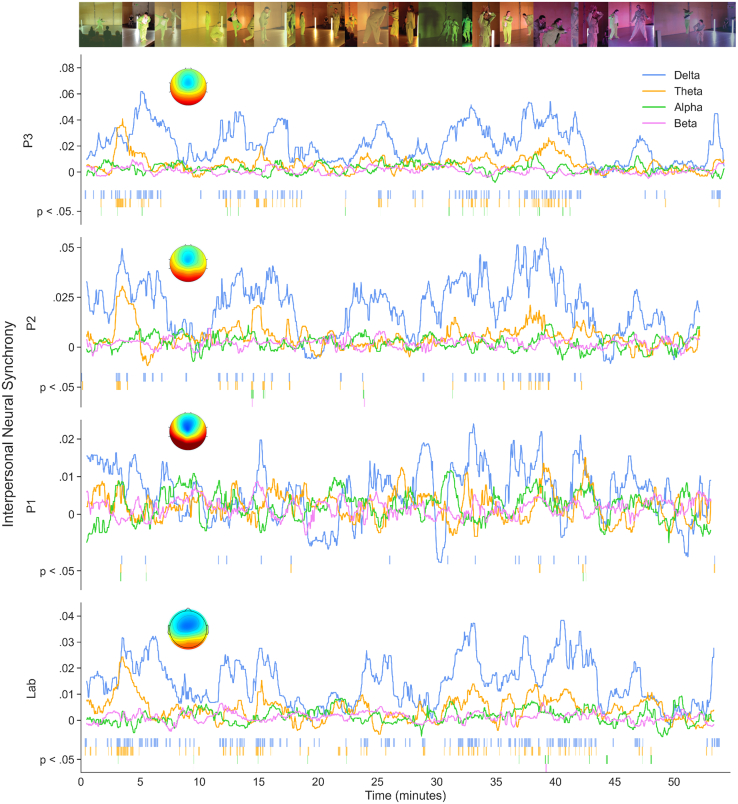
Time- and frequency-resolved correlated components analyses (Delta, Theta, Alpha, Beta) for live performances P1, P2, P3, and the laboratory condition where individual viewers watched a video of P3. CCA time series are smoothed by selecting the median value in 60-s windows Vertical lines indicate time-points (seconds) when C1_delta_ is greater than the 95^th^ percentile of INS calculated from 1,000 randomized datasets INS was most pronounced in the delta band across all three performances and the lab condition. Topographical plots indicate the spatial distribution of synchrony component C1_delta_. Red/blue colors indicate the direction of correlation (positive/negative) with C1_delta_ and EEG activity in that region.

For each performance (P1, P2, and P3), we extracted the first three correlated components (hereafter C1, C2, and C3). These components reflect correlations in EEG activity between participants across time that maximize the ratio of between-to within-participant covariance. Time-resolved CCA was computed in five-second windows with an 80% overlap for the entire duration of the performance. Across all performances, C1 captured the highest amount of covariance in audience frequency-band EEG activity (beta, alpha, theta, delta; Figure 3). Cluster-based permutation tests identified significant regions of timepoints in the four frequency-bands relative to the 95^th^ percentile of synchrony values from corresponding randomized EEG datasets. Across all performances, components in the delta-band showed the highest number of significant clusters (C1_Delta_; P1: 17 clusters, P2: 40 clusters, P3: 91 clusters). The length of clusters in seconds was also greater for the delta frequency band (see Table S2). A chi-squared test of independence for each performance showed a statistically significant association between frequency band and number of significant clusters for Component 1 (P1: χ^2^ (3, 3216) = 21.83, *p* = 0.0001; P2: χ^2^ (3, 3160) = 70.31, *p* < 0.0001; P3: χ^2^ (3, 3283) = 170.13, *p* < 0.0001). Pairwise χ^2^ tests of significant timepoint clusters between all four frequency bands indicated more significant time-points for delta compared to other frequency bands (see Table S3). Component 1 in the delta band (C1_delta_) also explained the most covariance across components and frequency bands. Overall, in Performance 1 (P1), C1_Delta_ explained 26.82% of the group covariance, C2_Delta_ explained 13.43%, and C3_Delta_ explained 11.32%. This pattern was replicated across both P2 (C1_Delta_ = 41.56%, C2_Delta_ = 9.93%, C3_Delta_ = 7.1%) and P3 (C1_Delta_ = 41.92%, C2_Delta_ = 10.07%, and C3_Delta_ = 8.62%).

To provide an even stronger control for spurious correlations not linked to the unfolding performance, we collected active eyes-open Resting-State (RS) EEG from audience members immediately before they entered the performance space (P1: *n* = 15/20, P2: *n* = 15/18, P3: *n* = 17/21). RS data were recorded individually at the end of EEG preparation in a shared space. Repeating 2.5-min segments of RS data were then used as a baseline for C1_Delta_ calculated on segments of the same duration for the whole performance. For P3, 9 out of 21 choreographic sections were statistically significant (Wilcoxon signed-ranks, *p*_*FDR*_ < 0.05), with higher C1_delta_ for the performance compared to C1_RS_ Figure S1. For P2, segment 16 was higher for the Performance than for RS (*p*_*FDR*_ = 0.023; (gray shaded sections in Figure S1A)). P1 showed the opposite pattern with the C1_RS_ > Performance for all segments (*p*_*FDR*_ < 0.05), due to unexpectedly high INS_Delta_ in the baseline condition. In sum, while INS was most prominent in the delta frequency band across all three performances, P2 and P3 emerged as the performances with the highest synchrony, relative to both circularly shifted and resting state data. These results are in keeping with measures of data quality and synchronization (see Data/Methods S1 and Figure S7).

While the strength of INS relative to both baseline measures varied across the three performances, we did not observe any differences in summative audience engagement immediately after viewing. Audience members were generally engaged and enjoyed watching all three performances (*N =* 57, data missing for two participants). Twenty-eight questionnaire responses were given on a Likert scale of 1–7 from Disagree to Agree (e.g., “*I enjoyed the performance”*: *M = 5.14, SD =* 1.48, “*I was absorbed in the performance”*: *M =* 5.01, *SD =* 1.58, “*The performance held my attention,” M =* 4.72, *SD =* 1.58). None of the questionnaire ratings differed between performances (*p*_*FDR*_ > 0.91).

### Artistic intentions align with continuous audience engagement

To test if watching dance indeed involves communication between dance artists and the audience, we computed correlations between the artist’s continuous ratings of predicted audience engagement and actual engagement ratings from viewers in an independent online study, based on a video of the third performance of *Detective Work* (https://youtu.be/RivFBmqJxzA).

We collected ratings from three core members of the artistic team: the choreographer, the second performer, and the dramaturg. In the context of contemporary dance works like *Detective Work*, one of the dramaturg’s tasks is to emulate the audience’s point of view, anticipating and shaping how artistic intentions are realized during the creation process. This is particularly important for *Detective Work*, since the choreographer also performs in the work himself and therefore cannot experience the work from a 3^rd^ person perspective. We therefore predict positive correlations between the predicted engagement ratings of the choreographer and the dramaturg, as well as between the dramaturg, the choreographer, and spectators of *Detective Work*. Alignment between intended and actual engagement would indicate effective performer-spectator communication.^13^^,^^51^

**Figure 4:**
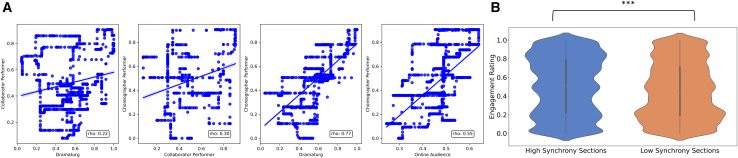
Continuous engagement ratings (A) Scatterplots of relationships between artistic team and online audience continuous ratings with Spearman’s rho reported The ratings of the Choreographer and the Dramaturg showed the highest correlations. The dramaturg’s role during the creative process is to emulate the audience’s perspective. (B) For P3, performance sections with delta band synchrony significantly higher than in the resting state were also continuously rated as more engaging by an independent online sample matched for dance experience. Violin plots depict the distribution of ratings, with inner boxplot and median value. Wilcoxon-signed rank test; ∗∗∗*p* < 0.0001.

Comparing all three members of the artistic team, a correlation matrix of these ratings with a median rolling window of 180 s (2.5 min) showed that the choreographer and dramaturg (rho = 0.77) provide more similar ratings than the choreographer and the performer (rho’s = 0.22, 0.30; all p’s < 0.0001). Additionally, we collected continuous ratings of engagement from a separate online sample watching the video of performance 3 (*N* = 23, to match the sample size of the live EEG audience and scoring high on observational dance experience (M > 5)). The median online audience ratings of actual engagement correlated significantly with the choreographer’s ratings of predicted engagement (rho = 0.55, *p* < 0.001), see Figure 4A for correlations between actual and predicted engagement.

Finally, performance sections with C1_Delta_ significantly higher than the resting state (Perf. > RS) were also more engaging to watch (M = 0.46, SD = 0.30) (Figure 4B) compared to sections not significantly different from the resting state (Perf. = RS; M = 0.51, SD = 0.31; Figure S1A). This comparison was statistically significant across all timepoints (z = 261995013.0, *p* < 0.0001), with Perf. > RS showing higher engagement ratings (Mdn = 0.52, SE = 0.002) compared to Perf. = RS seconds (Mdn = 0.43, SE = 0.002).

Across all three performances (see Figure S1B), and in keeping with the analysis of memorable moments above, C1_Delta_ was highest in one specific section of the choreography where both performers interacted with the audience through a direct and sustained exchange of gaze (“Unison” section). C1_Delta_ during the Unison section is also significantly higher than during the resting state for two out of three performances. Together our findings show that engagement across all three performances reflects dynamic artistic intentions that were shaped during the creative process of *Detective Work*. Moreover, choreographic sections with high and low predicted and actual engagement ratings map onto sections with higher and lower delta band INS, respectively, and reflect social interactions between performers and spectators.

### Artistic intentions and performance features predict delta band interpersonal neural synchrony

To better understand the relationship between specific aspects of the performance, audience experience, and the time course of C1_Delta,_ we quantified several continuous audiovisual features of the performance to predict INS over time. Performance features include spectral power and beat clarity of the soundtrack (extracted from the video), stage lighting (recorded from a light diode on stage), movement acceleration (recorded from wrist accelerometers) and distance between dancers on stage (extracted from a camera filming the stage from the ceiling; see STAR Methods), and respiration synchrony of the audience members (calculated using the same CCA method). Except for movement acceleration, spectral power for all performance features was well outside the delta frequency band (see Figure S2). We used univariate Granger causality analyses to identify pairwise predictive relationships between these performance features and C1_Delta_. The same analysis was applied to the randomized brain synchrony component (C1_random_) based on 1,000 repetitions of randomized circularly shifted EEG data, for each performance. A univariate approach is more appropriate for our data, as it takes into account the qualitatively different nature of our performance predictors, which could affect INS at different timescales. Multivariate approaches optimize the relative contribution of many predictors at a specific time-lag and are typically used to model temporal relationships of brain activity recorded from different electrodes or brain areas. Conceptually, the multivariate nature of individual performance features is captured by the choreographer’s predictive ratings of engagement.

**Figure 5:**
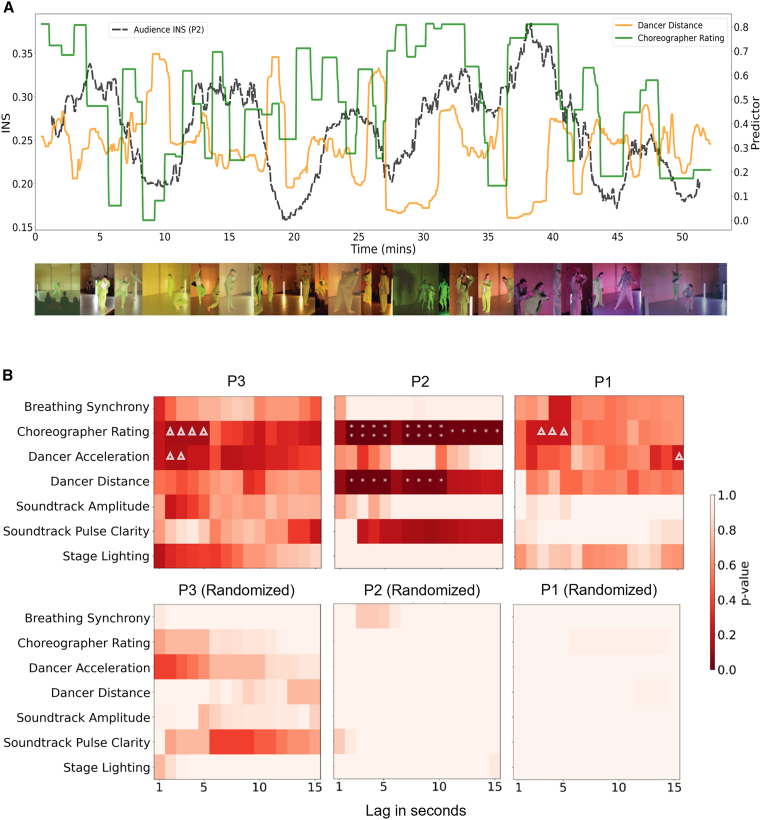
Relationships between performance features and audience interpersonal neural synchrony (A) Time-course of audience INS_Delta_ for P2 with significant Granger-causal variables, Choreographer Rating (green) and Dancer Distance (orange), across the entire performance duration. A moving median 60-s window is applied to time-series for visualization purposes. (B) Heatmaps depict FDR corrected *p*-values from pairwise Granger causality tests with interpersonal neural synchrony INS_Delta_ as the outcome variable for Performances 1, 2, and 3 (P1, P2, P3). ∗∗ *p*_*FDR*_ < 0.01, ∗ *p*_*FDR*_ < 0.001, Δ *p*_*uncorrected*_ < 0.01. All performance features for each of the three live performances are viewable as an interactive plot from: laura-rai.github.io/neurolive-DW/index.html.

All performance feature time-series and the choreographer’s predicted engagement ratings met the condition of stationarity according to the Augmented Dickey-Fuller test (*p’s* < 0.05), except for stage lighting, which was differenced prior to analysis. Granger Causality tests with C1_Delta_ or C_random_ as the outcome variable were performed for each individual predictor time-series from one to 15-s lags. Across all three performances, the choreographer’s ratings of the audience’s collective attentional engagement were the most significant (*p*-value) and most consistent (number of significant lags) predictor of audience brain synchrony. Following FDR correction for multiple pairwise comparisons, the performer’s ratings for P2 emerged as the strongest predictor and was significant across lags 2–5 (*p*_*FDR*_ < 0.01) and 7–15 (*p*_*FDR*_ < 0.05), followed by Dancer Distance from lags 2–5 and 7–10 (*p*_*FDR*_ < 0.05) (see Figure 5). Before correcting for multiple comparisons, P1 and P3 showed a relationship between INS and Choreographer Performer Rating (P1: lag 4, *p* = 0.003, lag 5, *p* = 0.008, lag 3, *p* = 0.008, and lag 2, *p* = 0.02; P3: lag 4, *p* = 0.001, lag 3, *p* = 0.004 at, and lags, 2, 5, *p* = 0.005), followed by Dancer Acceleration (P1: lag 15, *p* = 0.009; P3: lag 2, *p* = 0.008; lag 3, *p* = 0.009; lag 7, *p* = 0.01). Across all three performances, tests with C1_random_ showed no statistically significant results compared to the true analysis (distributions of *p*-values for C1_delta_ and C1_random_ are presented in Figure S3).

Greater INS could result from tighter alignment of spectators’ brains to the unfolding performance features (phase synchrony), heightened arousal (amplitude synchrony), or a combination of both. However, Granger-causality tests with mean time-resolved audience delta- and alpha-band EEG power for P3 as the outcome variable (instead of C1_delta_) and the same performance features as predictors showed no significant relationships before corrections for multiple comparisons (all *p’s* > 0.9). Further, there was no granger-causal relationship between EEG power and C1_D__elta_ (*p’s* > 0.13). This indicates that mean EEG power is not sensitive to the choreographer’s intentions in the same manner as audience INS.

Delta-band activity is associated with breathing patterns; however, an additional set of Granger Causality tests with Respiration Synchrony as the outcome, and the choreographer ratings and performance features as Granger-causing predictors showed no significant relationships from lags of 1– 15 s (uncorrected *p’s* > 0.06).

### Delta-band interpersonal neural synchrony is reduced if people watch a video of the performance alone in the lab

**Figure 6:**
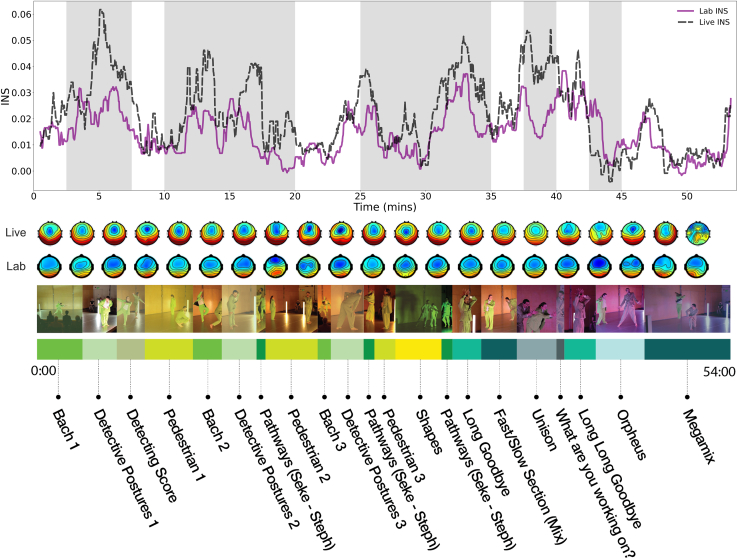
Comparison of INS for the experience of P3 live or in the lab Highlighted segments in grey indicate Live Performance INS_Delta_ > Lab INS_Delta_, except for the final highlighted segment when Lab INS_Delta_ > Live INS_Delta_ (*p*_*FDR*_ < 0.05). Group comparisons between laboratory and live conditions were conducted on 2.5-min sections of the performance. The analysis pipeline was identical for laboratory and live data analysis. Viewers were matched for dance experience. Topographical plots indicate the spatial distribution of synchrony component C1_D__elta_ for each 2.5-min segment in the live and laboratory conditions.

Audience brain synchrony might not only depend on engagement with the dynamic features of the performance but also on the live performance context. In a lab study where individual audience members watched a video of P3 on their own, INS_Delta_ was reduced relative to experiencing P3 live (see Figure 6). Applying the same analysis pipeline as for the live performances, we found that the delta frequency band also explained the greatest amount of variance between individuals in the lab setting (C1_Delta_ = 40.12%, C2_Delta_ = 11.29%, C3_Delta_ = 10.82%, see Figure 3), with 108 statistically significant timepoint clusters based on the 95th percentile of CCA values from randomized data. Importantly, mean audience C1_Delta_ during the live performance (*M =* 0.03, *SD =* 0.03) was significantly higher than for the lab setting (*M =* 0.02, *SD =* 0.02; *t*(6458) *=* −13.95, *p* < 0.0001), but across both live and lab contexts the temporal structure of C1_Delta_ was preserved. Moreover, the difference between live and lab conditions was most pronounced for the most engaging sections (group comparisons of C1_Delta_ between live and lab showed 12/21 significant segments *p*_*FDR*_ < 0.05; shaded segments in Figure 6). For 11 segments, INS_Delta_ > Lab INS_Delta_, whereas in one segment (42.5–45 min) the Lab INS_Delta_ was significantly higher than Live INS_Delta._ Experiencing the dance performance live and together was therefore associated with larger differences in brain synchronization between audience members over the course of the performance.

### Amplitude and phase-dependent EEG correlates of watching dance together

Comparing the live performances to the lab study does not dissociate social liveness (experiencing *Detective Work* alone vs. together) from physical liveness (watching the live show vs. watching a video recording, see Figure 1D and Table S1). Moreover, CCA does not separate phase and amplitude-based audience brain synchrony. We computed delta phase locking value (PLV_Delta_) and alpha/delta EEG power for the most and least engaging sections for all live performances (live group), the cinema screening (recorded group), and the lab study (recorded group) to dissociate the influence of dynamic performance content (high vs. low engagement) from the influence of performance context (live vs. screening vs. lab).

A linear mixed effects model on pairwise PLV_Delta_, with continuous engagement (high versus low), performance context (live, cinema and lab), region (occipital versus central) and their respective interactions as fixed factors showed that delta phase among spectators was more tightly coupled during high engagement sections (β = 0.03, t = 12.41, *p* < 0.001), particularly across occipital regions (β = 0.012, t = 2.43, *p* = 0.02). There was a significant interaction between engagement and performance context for Lab versus P3 (β = 0.01, t = 2.23, *p* = 0.03), Lab versus Cinema (β = -0.03, t = 4.55, *p* < 001), and marginally for Lab versus P2 (β = 0.01, t = 1.96, *p* = 0.05), with higher PLV_Delta_ for the live and cinema screening audiences during high engagement sections compared to the Lab, and vice versa for low engagement sections (see Figure S5), but no significant differences between cinema screening and the live performances. The intra-class coefficient for random effects suggested a small influence of participant pair (max. ICC = 0.027). Pairwise distance of seating positions between audience members did not affect PLV_Delta_ (see Figure S5).

A linear mixed effects model with EEG power as the outcome variable, and frequency band (delta, alpha), engagement (high vs. low), and performance context (live, lab and cinema), and their respective interactions as fixed factors revealed that delta was higher than alpha power (β = 0.77, t = 10.59, *p* < 001) but there was no main effect of engagement. Both frequency bands were higher in the lab (recorded-alone) than during the cinema screening and the live performances (live-together and recorded-together, P1-Lab (β = −0.58, t = −2.2, *p* = 0.03), P2-Lab (β = −0.79, t = −2.91, *p* = 0.004), and Cinema-Lab (β = −0.58, t = −2.04, *p* = 0.02), but did not differ between live performances and cinema screening (see Data/Methods S1 for full analyses of PLV and EEG power). In sum, both PLV_Delta_ and alpha/delta EEG power were sensitive to social context, that is, either higher (Delta PLV) or lower (alpha/delta power) when dance was watched together live or in the cinema than alone in the lab.

## Discussion

Our study reveals a neural correlate of continuous audience engagement with dance that is sensitive to both performance content (artistic intentions and dance movement) and performance context (social/physical liveness). Across three live dance performances, we show that inter-brain synchrony among groups of audience members was greatest in the delta frequency band (1–4 Hz). Dynamic fluctuations in audience brain synchrony track the dancers’ movements, the choreographer’s intentions, and continuous audience engagement. A close relationship between predicted and actual engagement ratings shows that experiencing dance indeed involves communication between performance makers, performers, and spectators.^22^^,^^23^^,^^51^^,^^52^^,^^53^ Immediately after watching the dance performance, engagement, curiosity, and felt connection with the performers were highest when spectators experienced dance live and together with others, in line with an evolutionary origin of live dance and music as participatory performances and collective rituals^19^^,^^20^ for social bonding and signaling.^12^^,^^15^^,^^16^^,^^21^^,^^22^^,^^54^ Watching dance evoked a synchronized brain response in the audience, which was most pronounced among co-present spectators, but did not necessarily differ in trajectory between the experience of live and recorded versions of the dance performance. In other words, watching dance together and live heightened people’s engagement with the performances and their felt connection with performers, but effective communication of non-verbal expressive intentions through movement^13^^,^^27^^,^^55^^,^^56^ occurred during both live and screened versions of *Detective Work*.

### Delta band and joint attention

What drives brain synchrony in the delta-frequency band? On the one hand, existing joint attention EEG studies in education^47^^,^^57^ or music,^58^^,^^59^ suggest neural correlates primarily in the alpha band.^60^ On the other hand, fMRI studies on brain synchrony while watching long, continuous and often narrative videos or films alone in the lab reveal neural correlates of engagement that occur at very slow frequencies, below 1 Hz.^37^^,^^61^^,^^62^^,^^63^^,^^64^ Instead, our study reveals fluctuations of audience brain synchrony between 1 and 4 Hz as an important index of continuous engagement.

Delta band activity is associated with a range of cognitive processes, including increased mind-wandering,^65^^,^^66^^,^^67^ neural alignment to sentences, music or movement phrases,^68^ rhythm or memory-based temporal prediction,^69^ watching dance with music,^70^ and is sensitive to social information, including faces.^71^ Higher delta-band power is also associated with default mode network activity,^72^ which in turn has been linked to self-relevant or moving aesthetic experiences for visual art^73^^,^^74^ or when watching dance.^75^ We found that delta band INS was greater in occipital regions and was predicted by movement acceleration and distance between dancers. Audience brain synchrony thus reflects greater attentional alignment to the dancers’ movements. If dancers are positioned closer together, the audience’s attention is likely focused toward them, which is consistent with activations of the lateral occipital gyri and surrounding regions of the action observation network when observing social interactions in dance.^75^^,^^76^^,^^77^ Delta-band INS as a correlate of individual or joint audience attention to the dancer’s movements is also consistent with comparable levels of audience brain synchrony across different seating positions and watching *Detective Work* together, either live or in the cinema.

### Nonverbal communication between dancers and spectators

The consistent time course of delta band INS is particularly striking given that (a) *Detective Work* does not provide a linear narrative that audience members can easily follow (b) dance movements are partly improvised and (c) do not align to a musical beat. Nonetheless, we can replicate the same pattern of results across three independent live performances. Moreover, we show that engagement with *Detective Work* cannot easily be reduced to single features of the performance, such as the unfolding musical score or the lighting design. Rather, as conceptualized in early empirical accounts of aesthetic experience (e.g., Fechner, 1876),^78^ it is the multi-layered combination of all elements of the live performance event that underlies audience engagement and is best captured by the artist’s continuous prediction of continuous engagement.

Delta INS and continuous engagement were highest during moments of direct performer-spectator interactions, that is, when performers made direct eye contact with individual members of the audience (Unison section). Importantly, within the context of the entire performance, the Unison section is characterized by ambient sound and extremely slow non-rhythmical movements, further supporting the idea that it is the socially interactive dimension of this section that drives audience engagement and not the saliency of low-level visual or auditory performance elements. Interestingly, engaging audience members via direct gaze breaking the fourth wall – was effective across performance contexts, although lower when a dance recording was watched alone. Breaking the fourth wall is a widely used performative device to engage audiences in theater,^79^^,^^80^^,^^81^ stand-up comedy,^82^^,^^83^ live music concerts^29^^,^^82^^,^^84^ and on television, suggesting that direct social interactions are a powerful tool to engage audiences across live and screened performances.

### The role of performance context: Live vs. screening vs. lab

**Figure 7:**
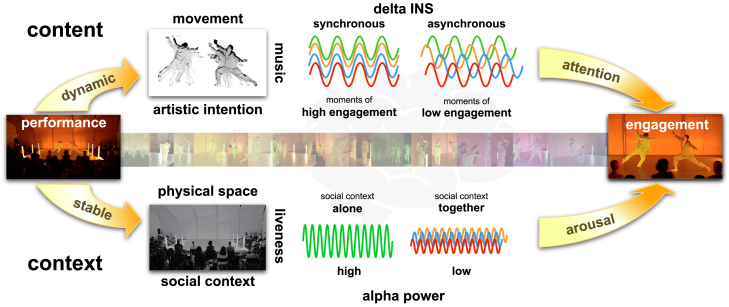
A model of EEG correlates of continuous and summative engagement with live dance performances that proposes distinct neural correlates for tracking performance content (delta INS) and performance context (alpha/delta power)

Existing research on brain synchrony and engagement with dance, music or film typically involves recorded stimuli, including TV shows or ads,^85^^,^^86^ political speeches,^42^ music,^87^ film or movie clips,^37^^,^^39^^,^^40^^,^^61^ or speech^88^ but do not involve any actual experience sharing between people, or co-presence between performers and spectators. Based on our findings we propose a model of engagement with dance performances (Figure 7) that distinguishes between performance content and context on the one hand, and continuous and summative engagement on the other hand. Delta INS reflects artistically directed, dynamic individual or joint attention to the performance content, modulated by performance and social context. Collectively viewing dance produces larger differences between moments of focused and dispersed joint attention by increasing audience arousal levels.

We acknowledge that there are large stimulus differences between lab, cinema, and live studies and between high and low engagement sections, yet we argue that it is the presence of other people that appears to drive differences between performance contexts, for both moments of high and low engagement alike. Although we do not directly compare the effect of watching the edited and the unedited video collectively, the relatively lower summative engagement in the studio screening compared to the cinema screening suggests that the purpose and effect of video editing may be to mimic the live experience, potentially synchronizing Delta PLV during high engagement sections and desynchronizing it during low engagement sections.

### Brain activity, head, and eye movements

It is possible that delta band synchrony in our study partly reflects synchrony in people’s gaze and heading direction or other movement artifacts; however, after pre-processing, very few outliers and only up to 3% of data needed to be excluded from the INS analysis (see STAR Methods). Madsen and Parra^44^ showed that saccade rate, but not head velocity, is coupled with EEG activity, and correlates between individuals watching videos sequentially in a laboratory. Similarly, Griffiths et al.^89^ showed that ERP correlates of head movement direction with a latency of ∼1 s are dissociable from electro-muscular activity and acceleration of the head. While our experimental set up does not allow us to completely remove the influence of head or eye movements, we clearly show that delta band INS is meaningfully related to the dancer’s movements and to predicted and actual engagement. Any contribution of the spectator’s movements to our findings is therefore not simply an artifact but reflects synchronized audience behavior that is aligned to the unfolding performance. Moreover, we can replicate the temporal structure of delta band INS across three live performances, in which people’s eye and head movements will vastly differ between seating positions. Yet, shorter physical distance between seating positions does not result in greater INS. Most importantly, however, we replicate our findings in a controlled lab environment in which any movement is minimal as participants watch a video on the screen.

### Conclusion

Our study presents a novel and ambitious interdisciplinary approach to studying the evolutionary functions and origins of the performing arts by combining artistic and scientific co-production with large-scale real-world neuroscience studies and a series of carefully controlled follow-up studies in different contexts. Distinct phase and amplitude-based EEG correlates highlight the relative importance of performance content and context for engagement with dance. Our findings show that audience engagement is measurable as artistically directed brain synchrony in the delta frequency band aligned to the performers’ movements. Audience brain synchrony is most pronounced during social interactions between dancers and spectators and when dance is experienced together rather than alone. The evolutionary ancient social functions of participatory performances are thus relevant to and preserved in cultural engagement with live contemporary dance.

## Resource availability

### Lead contact

Requests for further information and resources should be directed to and will be fulfilled by the lead contact, Guido Orgs (guido.orgs@ucl.ac.uk).

### Materials availability

No new materials (i.e., reagents) were created in the process of this human study.

### Data and code availability

• Data: EEG data are available on the University College London Data Repository: https://rdr.ucl.ac.uk/articles/dataset/Dataset_of_mobile_EEG_recordings_from_audiences_watching_a_live_dance_performance_Detective_Work_/28508744/1.
• Code: Analysis scripts are available on Open Science Framework: https://osf.io/uz573.
• Additional analyses of the results are provided in Data S1/Methods S1: Tables S1–S4; Figures S1–S10, and analysis related to Figure 7.

## Acknowledgments

This research received funding from the 10.13039/501100000781European Research Council (ERC) under the European Union’s 10.13039/501100007601Horizon 2020 research and innovation program (grant agreement No. 864420 - Neurolive - PI GO, CO-I's JW, DCR, MS). We thank Alex Neidert for project coordination, Iris Chan for artistic production, and those involved in the artistic creation of *Detective Work*: Seke Chimutengwende (Choreographer & Performer), Stephanie McMann (Collaborator & Performer), Jamie McCarthy (Composer), Annie Pender (Costume Designer), Jackie Shemesh (Lighting Designer), Charlie Ashwell (Dramaturg), Helena Webb (Rehearsal Director), Eleanor Sikorski (Videographer and Filmmaker), and Hugo Glendinning (Photographer). Thank you those who assisted with EEG preparation**:** Azelie Koffi**,** Becky Chamberlain**,** Camila Pinto**,** Daniele Porricelli**,** Diana Omigie**,** Dwaynica Greaves**,** Emmanuel Mahieux**,** Friendred Peng**,** George Bird**,** Giuseppe Lai**,** Heijo van de Werf**,** Jan de Fockert**,** Jeff Schultz**,** Jose Van Velzen**,** Katerina Vafeiadou**,** Lynn Saad**,** Mahnoosh Allahghadri**,** Manuel Mello**,** Margherita Tecilla**,** Maria Del Carmen Herrojo-Ruiz**,** Merritt Millman**,** Mirko Febbo**,** Ruchi Wieder**,** Sarah Hashim**,** Sebin Sabu**,** Sonia Abad Hernando**,** Stacey Humphries**,** Vlada Trofimch. Thank you to Adam Garrow for assistance with questionnaire transcription. Thank you to Federico Calderon for assistance with photoresistors.

## Author contributions

Conceptualization: GO, MS, JW, DCR, LR, and HL. Data curation: LR, HL, EB, CT, and SAH. Formal analysis: LR and EB. Funding acquisition: GO, MS, JW, and DCR. Investigation: LAR, HL, MS, JW, and GO. Methodology: LAR, HL, MS, JW, GO, DV, and CT. Project administration: LAR, HL, MS, and GO. Resources: LAR, HL, MS, JW, GO, and MWF. Validation: CT and EB. Visualization: LAR and HL. Writing – original draft: LAR and GO. Writing – review and editing: All authors.

## Declaration of interests

The authors declare no competing interests.

## STAR★Methods

### Key resources table

**Table:** 

REAGENT or RESOURCE	SOURCE	IDENTIFIER
**Deposited data**		

University College London Research Data Repository	This paper	https://rdr.ucl.ac.uk/articles/dataset/Dataset_of_mobile_EEG_recordings_from_audiences_watching_a_live_dance_performance_Detective_Work_/28508744/1

**Software and algorithms**		

MATLAB R2021b/2023a	Mathworks	https://www.mathworks.com
Correlated Components Analysis	Parra et al.^45^	https://www.parralab.org/isc/
Jamovi v2.2.5	Jamovi	https://www.jamovi.org
Statsmodels v0.13.5		https://www.statsmodels.org/v0.10.2/index.html
Gorilla Experiment Builder	Anwyl-Irvine et al.^90^	https://www.gorilla.sc
Prolific Academic	Prolific	https://www.prolific.co
EEGLAB	Delorme and Makeig^91^	https://eeglab.org/

**Other**		

Custom analysis scripts	This paper	https://osf.io/ursmc/

### Experimental model and study participant details

For the live performances, participants (total *N* = 69) were recruited via the Siobhan Davies Studios website by purchasing a ticket for the performance event *Detective Work* and opting-in to participate in the EEG/wearable sensors component of the study. The final EEG sample after data exclusion was 59 (P1, *n* = 20; P2, *n* = 18; P3, *n* = 21). Nine datasets were lost due to technical errors, and one dataset was rejected due to loss of LSL markers/photoresistor signal. All participants provided informed consent prior to taking part in the study as approved by the Research Ethics and Integrity sub-committee of Goldsmiths, University of London and University College London (protocol number ICN-VW-03-092024A).

The final live performance EEG audience samples’ age (P1: *M =* 43.27, *SD =* 16.79; P2: *M =* 34.13, *SD =* 16.47; P3: *M =* 33.94, *SD =* 9.09), and dance experience (Gold-DSI; Participatory (P1: *M =* 5.41, *SD =* 1.1; P2: *M =* 5.26, *SD =* 1.55; P3: *M =* 5.24, *SD =* 1.29) and Observational (P1: *M =* 5.25, *SD =* 1.18; P2: *M =* 4.92, *SD =* 1.63; P3: *M =* 5.37, *SD =* 1.06)) did not differ between performances (H = 4.5, *p* = 0.12; H = 0.39, *p* = 0.82; H = 0.53, *p* = 0.77). Gender was reported as follows: P1: 13 female, 4 male, 1 non-binary; P2: 6 female, 4 male, 4 non-binary, 1 other; P3: 10 female, male, 1 non-binary, 1 other.

The cinema screening was conducted at Goldsmiths University and comprised three showings of the edited video version of Performance 3 to three independent audiences. Overall, 57 participants (38 female, 3 non-binary, 13 male) with a mean age of 30.83 (*SD =* 11.56) and completed the same questionnaire as the audiences of the live performances. The cinema group reported lower dance experience compared to the live audience (Participatory: *M =* 4.54, *SD =* 0.95, U = 520, *p* < .001; Observational: *M =* 4.19, *SD =* 0.97; U = 448, *p* < .001). Some devices in the cinema screening did not receive Lab Streaming Layer markers, or the photoresistor signal was too noisy to align recordings, resulting in a reduced EEG sample size (N = 28; Screening 1 n = 7; Screening 2 n = 13, Screening 3 n = 8; see Figure S7C).

The studio screening was conducted at Siobhan Davies Studios on the 17^th^ November 2024. 24 participants (18 female, 1 non-binary, 5 male) were recruited via Siobhan Davies Studios, social media, and via UCL with a mean age of 34.08 (*SD =* 12.65), and were matched for dance experience with the live audience group (Participatory: *M =* 5.09, *SD =* 0.91, U = 718, *p* = 0.17; Observational: *M =* 5.34, *SD =* 1.08, U = 584.5, *p* = 0.86). They completed the same questionnaire as the previous groups. All EEG data for the studio screening were lost due to a data streaming issue.

The laboratory-based experiment was conducted at the Max Planck Institute for Empirical Aesthetics in Frankfurt am Main, Germany. The participants (*N* = 28) were matched to the EEG participants from the live Performance 3 in terms of their age (U = 291, *p* = 0.96) and previous dance experience (PDE: U = 256.5; ODE: U = 285.5, *p* = 0.06), resulting in a comparable sample (age: M = 39.43, SD = 19.9; PDE: M = 5.0, SD = 0.9, *p* = 0.24; ODE: M = 4.9, SD = 1.0). Each participant (22 female, 6 male) provided written informed consent before taking part in the study, which was approved by the Ethics Council of the Max Planck Society.

### Method details

Information sheets explaining the background and procedure of the study were provided on the booking website and attached to an online questionnaire sent to participants in advance of the performance. Advertising material for the performance events were presented in posters on the Goldsmiths University campus, other universities, and arts/theatre venues in the London area. The event was also advertised online via social media promotions and mailing lists.

*Detective Work* was performed across three evenings to three different audiences. Each evening, 23 EEG participants and 18 non-EEG audience members had booked tickets to the event. EEG participants were allocated an arrival time for set-up on the performance evening (18:00, 18:30, or 19:00), with the performance scheduled to take place from 19:30 to 20:30. The set-up procedure took place in a dedicated room with 7–8 other participants at a time. EEG setup took approximately 30 minutes and was performed by two research assistants at a time (see Figure S8), including (i) the completion of a pre-performance questionnaire, (ii) EEG preparation/impedance check, (iii) application of the respiration belt, (iv) a short resting-state recording, and (v) the assembly of the equipment (amplifier, tablet, Sensebox) in a backpack.

After EEG setup, spectators were seated in the performance space in a staggered manner and in pairs, according to their arrival time and were provided with a box beside their chair to hold the EEG backpack. Following the performance, participants were instructed to remain in their seats to complete a post-performance questionnaire. Each performance was attended by roughly equal number of spectators wearing EEG and spectators without EEG.

For the cinema and studio screening experiments, audiences were seated in tiered rows in front of a large screen on which the edited (cinema) or unedited (studio) video of Performance 3 were projected, respectively (see Figure S8). Following the screening, participants competed the post-questionnaire.

For the lab-based EEG experiment, participants were seated approximately 70 cm from the screen in an electrically shielded EEG chamber with dimmed lighting and watched the unedited video of P3. The video, captured from a static wide-angle perspective, was presented in its full length using PsychoPy software. EEG data were collected using a Brainproducts system equipped with 64 active electrodes placed in the 10/10 international placement system. The EEG preparation was set up with an impedance of less than 20 kOhm for each electrode and recorded at 1000 Hz. Participants were able to adjust the volume of the loudspeakers themselves before starting the experiment.

#### Materials

##### Detective Work

*Detective Work* is a contemporary dance performance commissioned for this research project^48^ (a full length edited video of P3 is available at: https://youtu.be/RivFBmqJxzA). It was created and performed by choreographer Seke Chimutengwende in collaboration with dance artist Stephanie McMann and investigates choreography as a process of making and solving mysteries. The audiences encounter two performers wearing suits that can evoke associations with detectives as portrayed in television or film. Throughout the choreography, performers use notions of searching and re-searching as a source for both improvised and set movement material. The piece is arranged in 21 short choreographic sections (see Figure 6) that contrast markedly with each other in their atmosphere and movement dynamics, often abruptly shifting from one to the next. These choreographic sections are often repeated several times, but with slight variations or reconfigurations in loose analogy to the principle of counterpoint often employed in musical composition. The piece utilized set movement phrases, in addition to improvised sections, and theatrical techniques, such as making direct eye contact with the audience.

During two months of a co-productive creation process with the artistic and scientific teams we identified five specific performance features of particular research focus in *Detective Work*: the movement and location of the two performers on stage, the music soundtrack composed for the performance, as well as lighting design. We quantified these primarily audio-visual features for each of the three performances, in addition to continuous ratings of engagement and predicted attentional engagement, from a separate group of participants, the two dance performers (including the choreographer) and the dramaturg respectively, and audience breathing synchrony.

### Quantification and statistical analysis

#### Performance soundtrack

The performance soundtrack was composed and performed by Jamie McCarthy (available here: https://soundcloud.com/jamie-mccarthy-1/sets/detective-work). Acoustic and musical features of the performance soundtrack were automatically extracted using the MIR toolbox.^92^ Two features representing music dynamics and rhythm were extracted temporally using default windows and overlaps. Root mean square energy can be used as an index of perceived loudness and was computed by taking the root average of the square of the amplitude over 50ms windows with 50% overlap. Pulse clarity is an index of beat strength and was calculated over 5 second windows with 10% overlap. Data were interpolated to match the 1 Hz sampling rate of the remaining feature time-series.

#### Dancer movement acceleration and distance

Raw acceleration data in the form of x, y, and z coordinates were obtained from mBient sensors (MetaMotionR from MBIENTLAB INC, San Francisco, CA, USA) worn on the wrists of each performer. Data were first resampled to 20 Hz using the interpl function in MATLAB so that the difference in time between each datapoint was consistently 0.05s throughout the entire time-series. Movement acceleration was calculated for each wrist sensor by taking the root summed square, and combined acceleration was calculated from the median acceleration between the two performers.

The Euclidean distance between the two performers’ position on stage was calculated using the formula sqrt((personA_x - personB_x).ˆ2 + (personA_y - personB_y).ˆ2), where x and y refer to the coordinates of the dancers' position on the stage during each frame of a scene. These positions were obtained by a researcher manually tracking the centre point on the floor between each dancer’s feet using the recorded video. These coordinates were then adjusted to account for the mapping between the perspective of the camera and the floorplan. Data were interpolated and averaged over 5-second windows with 80% overlap to match the CCA calculations (Figure S9).

#### Performance lighting

Luminance was extracted by converting a wide-shot video of the live performances to grey scale and averaging pixel luminance for each frame. The luminance time-series was interpolated to match the 1-second resolution of the time-resolved inter-subject correlations.

#### Respiration acquisition

Respiratory activity was measured using a respiration belt placed on the abdomen at the point of maximal expansion during inhalation. The respiration belt was connected to the eego amplifier via an auxiliary channel of the Sensebox adapter and data were sampled at 500 Hz. The raw respiration belt signal was extracted for each subject and down-sampled to 250Hz, outliers outside a moving median of 0.5 second intervals were replaced with the surrounding average, and data were smoothed over a 1 second interval using a Savitzky-Golay filter (Power et al., 2020). Audience respiration synchrony was calculated using the same inter-subject correlations method as the EEG.^44^^,^^45^ (see below) .

#### Data synchronisation

A key consideration for our study was the synchronisation of EEG data for multiple participants outside a laboratory setting. Accurate time synchronisation between subjects is essential for offline data analysis of responses to naturalistic stimulus events. In the current study, EEG file recordings were timestamped with the Windows clock time when the recording was started. As Windows clocks drift across time and this can differ between devices, all devices were manually forced to synchronise with the Windows internet time server prior to each performance evening. Automated and manual markers were sent using Lab Streaming Layer (LSL) from a separate Windows laptop across a local network to all EEG devices. Each marker contained a unix timestamp of the local Windows laptop. Automated markers were sent every five seconds for the duration of the EEG recordings (see Figure S7A). Additionally, event markers denoting sections of the performance dramaturgy were manually sent by a member of the research team seated in the performance space.

As a second and independent method of data alignment, we synchronized all EEG using light sensors to a black out at the start and end of each performance. A custom-built photoresistor was connected to the auxiliary channel of the SenseBox adapter and attached outside each participant’s backpack. Finally, to align EEG data with the performer’s movements, EEG recordings were synchronised to the mBient sensors using the photoresistors and the onboard mBient light sensors before and after the performances (Figures S7A and S10). Change points in the photo-resistance signal at the onset of the first blackout were identified using the MATLAB function ischange. For each EEG recording, the first linear change in the slope of the photo-resistance signal was identified (i.e., the red circles in Figure S10B). Each participant’s data were then shifted towards the reference change point that minimised the difference between remaining change points.

#### EEG acquisition

Live performance EEG data were recorded using eego™ sports systems (ANT-Neuro, The Netherlands), with 32 Ag/AgCl channel wireless electrodes on Wavegaurd™ original caps. Electrodes were located according to the standardised 10/10 international placement system. Conductive gel (OneStep Cleargel) was applied in the electrode holders, using blunt tip syringes (ref for syringes). During EEG preparation, scalp impedances were monitored to be below 20 kΩ. EEG recordings were sampled at 500Hz, with CPz as the online reference channel. Data were acquired locally with eego system software (eemagine Medical Imaging Solutions GmbH, Berlin, Germany) on Windows Surface Pro tablets connected to each amplifier. Following data acquisition, the raw EEG data were exported to. cnt format.

Laboratory EEG data were recorded using a Brain Products EEG system with 64 active EEG electrodes on ActiCAPs. Electrodes were arranged in a 10/10 configuration and impedances were kept below 20 kOhm during preparation. The recordings were presented on a 24-inch computer monitor (BenQ, 144Hz, 24 inches, 1920x1080) with Neumann studio monitors in a sound-proof and electrically shielded EEG chamber. EEG recordings were sampled at 1000 Hz using BrainVision Recorder (version 1.23.0003) and EEG triggers were sent for later synchronisation.

#### EEG pre-processing

Existing multi-bran EEG studies almost exclusively use wireless systems with saline- or dry-electrodes, for example Chabin et al.^58^ use 14-channel systems, as do Dikker et al.^47^ Wet-electrode systems provide more signal reliability particularly in longer recordings, and in slower frequencies. Our systems additionally provide higher resolution (24 bit) and sampling frequency (here, 500 Hz), which improves the accuracy and temporal resolution of the recorded data. A comparison of mobile EEG systems using the Categorisation of Mobile EEG (CoME) Scheme, shows that eego sports was rated in the top 3 for system specification, which is a marker of high signal quality.^93^^,^^94^ For the Performance EEG recordings (∼50 minutes), data were first high pass filtered at 1 Hz. Next, data was down sampled to 250Hz. To eliminate artifacts corresponding to muscle and eye activity as well as other noise disturbances such as electrode contact loss, we made use of independent component analysis (ICA) (Comon 1994) as implemented in EEGLAB (default extended InfoMax). Independent components were inspected by a team of researchers (LR, SAH, HL) and were marked as artifacts based on a combination of topography, time-series, and power spectrum visualisations in EEGLAB, and the IC Label classifier.^95^ An average number of 16.25 (SD = 4.34) ICA components were removed from each dataset manually to keep the physiological integrity of the data (see https://osf.io/mkf4n). Compared to trial-based EEG studies, we reject a relatively large number of components per participants, due to the fact that over the course of an uninterrupted recording of 1 hour, some artifacts only occur a few times. A small number of bad channels were interpolated (*M =* 0.23*, SD =* 0.62). Next, data were low pass-filtered at 44 Hz, and then re-referenced to the average. As a validity check of data quality, the percentage of ‘good’ data was calculated using Artifact Space Reconstruction (ASR) by subjecting post-processed datasets to the clean_rawdata plugin in MATLAB. The percentage of data retained after removing ‘bad’ samples with 1 second windows and 66% overlap provided an indication of data quality, as described by Delorme et al.^96^ The mean percentage of good data was > 95% for each performance (P1: *M =* 96.32, *SD =* 3.04; P2: *M =* 95.72, *SD =* 2.74; P3: *M =* 95.25, *SD =* 3.71).

Following the procedure of Dmochowski et al.,^40^ EEG outlier samples were identified per dataset and channel as those exceeding four standard deviations of their respective channel. Outlier samples, along with 40ms segments before and after each outlier were set to zero values. The percentage of unique zero-value samples per performance and frequency band were very low (see Table S3). The duration of outlier data removed for the entire 55-minute performance was as short as 30 seconds and never exceeded 6 minutes in total (Percentage data removed across all three performances for delta *M (SD)* = 2.45% (1.03); theta = 3.22% (1.47); alpha = 5.66 (2.2); beta = 6.88 (2.07)).

Datasets were temporally aligned using two independent signals. First, using Lab streaming Layer (LSL) we used a regular 5s signal stream to mark a ‘heartbeat’ throughout the duration of the performance. This helps us to identify common points at the beginning and end of the performance. Secondly, signals from custom-made photoresistor circuits connected to each amplifier were used to align the data streams directly using common changes in lighting – specifically, the blackouts at the start of each performance. If LSL markers were not received by a device, the photoresistor signal was used as temporal reference point for aligning data - this was necessary for 8 data sets. For P1, 6 datasets were shifted so that the final maximum variability between photoresistor signal inflection points was 24 ms, for P2 the maximum variability was 54 ms, and for P3, 18 ms. These maximum offsets limit the effective sample rate, *f*, of two signals being compared, and according to Nyquist will dictate the maximum frequency of the signals as *f/2.* The largest offset (P2) equates to an *f* of 18 Hz, which means we can confidently assess synchrony across the delta, theta and low alpha bands for that performance. In dataset P3 however, with an *f* of 55 Hz*,* we can additionally study between-device synchrony across the alpha and low beta bands.

The pre-processing of the laboratory data followed the same steps as described for the live performances. During ICA, an average of 21.07 (SD = 4.65) components out of a total of 61 components per subject were rejected. Interpolation of the remaining bad channels was minimal (M = 0.07, SD = 0.26). For the following comparative analysis, the corresponding channels were selected (Fp1, Fp2, F7, F3, Fz, F4, F8, FC5, FC1, FC2, FC6, T7, C3, Cz, C4, T8, CP5, CP1, CP2, CP6, P7, P3, Pz, P4, P8, POz, O1, Oz, O2), resulting in a total of 29 channels. One electrode position was lost due to misalignment of the standard electrode placements between the two different systems used (Fpz is not available in Brain-Vision’s actiCAP 64-channel layout).

#### Brain synchrony: Correlated components analysis (CCA)

We chose group-based correlated components analysis (CCA^45^) to compute INS between all audience members across the entire duration of all three live performances, the lab study, and separately for delta, theta, alpha and beta frequency bands. INS was then compared to two baseline conditions. Firstly, against 1000 iterations of randomized, circularly shifted data, as recommended by Parra et al.^45^; such that component values for clusters of time-points outside the 95^th^ percentile of randomly permuted CCA analyses were considered statistically significant. Secondly, INS while watching the performance was compared to repeated segments of a 2.5-minute active resting-state control condition that we collected from each participant individually before the start of the performance using a signed-ranks test.

To investigate the temporal scale of INS between participants, EEG was bandpass filtered with a 5^th^ order Butterworth filter across four frequency bands (delta: 1 – 4 Hz, theta: 4 – 8 Hz, alpha: 8 – 12 Hz, beta: 12 – 20 Hz).

CCA components reflect linear combinations of EEG electrode activity that maximize correlations between participants. The first three components explain the highest amount of co-variance in EEG activity across time. Time-resolved INS was calculated in 5-second windows with 80% overlap. Code for this method is available from: https://parralab.org/resources.html and https://osf.io/69gfe). The CCA method was applied to data from each of the frequency filtered bands.

To correct for multiple comparisons across time-points and frequency bands when comparing time-resolved CCA to the randomised baseline, significant C1_Delta_ values of the true analysis outside the 95th percentile of randomized analyses were first entered into the Binary Connected Components ‘bwconncomp’ function in MATLAB. This identifies connected adjacent time-points (including contiguous points within a frequency band, and points in neighbouring frequency-bands) that exceed the 95th percentile threshold and clusters them together. A null distribution of clusters is calculated by taking the largest cluster of timepoints in each of the 1000 iterations. The 95th percentile of the null cluster distribution was then used as the basis for determining statistical significance of the true C1_Delta_ results, as depicted in Figure 3.

#### Granger causality analysis

To test whether audience synchrony components showed a relationship with performance features, a Granger Causality analysis was conducted (code available: https://osf.io/b62d7) with the statsmodels package.^97^ Each performance feature (Audio Pulse Clarity, Audio Root Mean Square, Choreographer Ratings, Dancer Acceleration, Dancer Distance, and Respiration Synchrony) was entered into a pairwise test with the audience brain synchrony component ‘C1_Delta_’ as the outcome variable. The same analysis was applied to the randomised brain synchrony component based on 1,000 repetitions of CCA analysis applied to circularly shifted EEG data. This was conducted for all three performances. We opted to use pairwise tests as they allowed us to test each performance feature at different time lags, for example we would not expect all features to Granger-cause C1_Delta_ at the same lag – as would be the case in a multivariate autoregressive model based on model fit.

#### Brain synchrony: Phase locking value (PLV)

While CCA provides a data-driven approach to identify a genuinely group-based correlate of shared engagement, it does not distinguish between phase- and amplitude-based synchrony, nor does it allow us to incorporate pairwise relationships between audience members into the analysis. PLV was calculated on band-passed EEG data in the delta frequency band for each time window *w* (each sample point at 250 Hz, for the entire duration of the performance) and pair of subjects *j* and *k* according to the equation below, adapted from^98^ where *θ*_*j*_ and *θ*_*k*_ denote the phase of the corresponding signals *s*_*j*_ and *s*_*k*_ (*j* /= *k*) at time *t*, m indicates the number of participants, *T* the size of *w*. The Hilbert transform was applied to extract the rising and falling of the signal, where the angle, i.e., the instantaneous phase, was taken. Then, the phase difference between the two time series data, which represents the locking of the two signals, was computed. Finally, these difference values were normalised into the PLV metric, by averaging over 5-second time windows with a 4-second overlap (i.e., consistent with the CCA method above).

*PLV*_*j,k*_ is a normalized index of synchronization with values in the range of 0 to 1, where 1 indicates perfect phase synchrony and 0 indicates none^99^^,^^100^. PLV_Delta_ was calculated for every possible pair of audience members for P1 (N_pairs_ = 190), P2 (N_pairs_ = 153), and P3 (N_pairs_ = 210), for each matching between-subject electrodes in occipital and frontal regions.

$$PLV_{j,k}=\frac{1}{T}\sum_{t=1}^{T}e^{i\left(\theta_{j}\left(t,w\right)-\theta_{k}\left(t,w\right)\right)}$$

Based on the CCA and continuous engagement ratings, PLV in the delta band for all live performances and the cinema screenings was extracted for two performance segments of interest: Pedestrian 1 (Low engagement) and Unison (High engagement), resulting in 3-minute segments for both (N_windows_ = 36, of 5-second windows for each 3 minutes). Based on the topography of CCA results, among 30 electrodes, six were extracted from the occipital and frontal regions: C3, C4, Cz, O1, Oz, O2 for the statistical analysis.

#### EEG power

Power spectral density was calculated using Welch’s method and Hanning window of length 250 points (i.e., 1 second) with 20% overlap for each participant averaged across channels. Alpha/delta EEG power was averaged within the same frequency regions as the INS analyses. For the linear mixed effects models analysis, outliers > 4 SD from the mean power were removed from analysis, this led to the removal of two participants’ data from the cinema screening sample.

#### Subjective engagement measures

##### Summative ratings of engagement

The post-performance questionnaire included questions adapted from Brown and Novak-Leonard,^101^ and others developed for the purposes of this study. These targeted factors such as enjoyment, captivation, social bonding, intellectual stimulation, emotional resonance, and a memory task. Responses were provided on a Likert scale ranging from 1 (Not at all/Disagree) to 7 (A great deal/Agree). Full details of the post-performance questionnaire are reported in Lee et al.^11^ and attached as a supplemental information. Questionnaire responses were non-normally distributed overall (Shapiro-Wilks tests for lab, live, cinema, and studio screening questionnaire responses *p’s* < .05, except for Items 10, 11, 17 (Lab), 17, 24 (Live), 3, 7, 19 (Cinema), 2, 5, 6, 11, 14, 19, 21, 24, 26 (Studio)), and showed homogeneity of variances between lab, live, cinema, and studio screening comparisons (Levene’s test *p’s* > 0.05, with the exception of Items 10 & 14). For the lab study, we used a German translation of the post–performance questionnaire. An analysis of the factorial structure of the post-performance questionnaire is reported in Lee et al., 2024.

##### Continuous ratings of predicted engagement

Both performers and the dramaturg provided continuous ratings while watching the video of Performance 3. The Choreographer-Performer additionally provided continuous ratings for Performances 1 and 2. They were instructed to rate the intensity of the performance, taking the perspective of an audience of the live performance. Here we defined ‘intensity’ as a measure of collective attentional engagement, as follows: “The intensity of the performance in this context relates to (your perception of) how different moments in the performance either more tightly or more loosely gather the audience’s attention into a state of shared focus. Moments of high intensity in the performance should synchronise the audience’s collective attention (tightly gathered), whereas moments of low intensity should produce more varied patterns of audience members’ individual attentional engagement over time (loosely gathered).” The rating experiment was created and hosted online on Gorilla.^90^ Ratings were extracted using online mouse-tracking, y coordinates represented the extent to which the performer expected there to be brain synchrony among the audience group (i.e., higher ratings = more expected synchrony). A moving window of 5 seconds with 80% overlap was applied to the continuous rating time-series for input to the Granger causality analysis.

##### Continuous ratings of actual engagement

A separate sample was recruited online to watch the Detective Work video and provide continuous ratings of engagement. Participants were recruited through Prolific (www.prolific.com) and were screened with the Gold DSI observational dance experience sub-scale to match the live performance audience. Participants with an ODE score > 5 were invited to participate in the rating experiment (*N* = 23; mean age = 36.3; 14 female, 9 male). They were instructed to move their mouse cursor up or down according to their engagement level: “When you are feeling engaged by the performance, move your mouse cursor higher on the screen. When you are not feeling engaged, move it down. By engagement we mean anything that makes you want to keep watching.”

#### Dance experience: Goldsmiths dance sophistication index (Gold-DSI)

The Gold-DSI was designed to assess dance experience, measuring individual differences in participatory and observational dance experience.^102^ The index consists of 26 items on a continuous scale, of which 20 items relate to general dance participation (body awareness, social dancing, urge to dance, and dance training; e.g., ‘I find it easy to learn new movements’), and a further 6 relating to observation (e.g., ‘I like watching people dance’). Responses are provided on a seven-point scale ranging from Completely Disagree to Completely Agree). The Gold-DSI factors have shown good to very good internal validity (alphas > .79). For the Lab study, we used a German translation of the DSI.

### Additional resources

Pre-registration of data collection and analysis: https://aspredicted.org/w9sy9.pdf.

## Supplemental information

Document S1. Post-performance questionnaire

Document S2. Figures S1–S10, Tables S1–S4, and Data S1/Methods S1
