# Supplementary material for: Delta-band audience brain synchrony tracks engagement with live and recorded dance
Source: iScience. 2025 Jul 7;28(7):112922. doi: 10.1016/j.isci.2025.112922 (PMC12432844; doi:10.1016/j.isci.2025.112922)
Supplement: Document S1. Post-performance questionnaire [file mmc1.pdf]

iScience, Volume *28*

Supplemental information

Delta-band audience brain synchrony tracks engagement with live and recorded dance

Laura A. Rai, Haeeun Lee, Emma Becke, Carlos Trenado, Sonia Abad-Hernando, Matthias Sperling, Diego Vidaurre, Melanie Wald-Fuhrmann, Daniel C. Richardson, Jamie A. Ward, and Guido Orgs

Post-Performance Questionnaire

Pseudonym Code:

Please rate how you are feeling right now.

Circle one number on each row corresponding how unhappy/happy and calm/excited you feel.

Unhappy 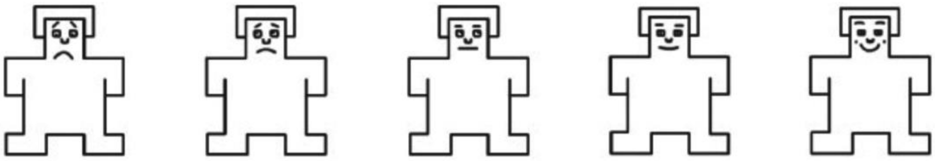 Happy

1 2 3 4 5 6 7 8 9

Calm 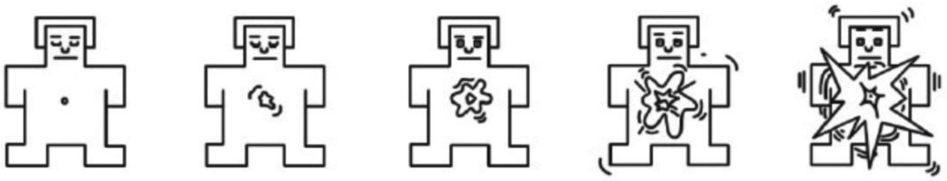 Excited

1 2 3 4 5 6 7 8 9

Please circle the appropriate number in response to the following questions.

1. I enjoyed the performance.

Disagree 1 -------- 2 -------- 3 -------- 4 -------- 5 -------- 6 -------- 7 Agree

2. I was absorbed in the performance.

Disagree 1 -------- 2 -------- 3 -------- 4 -------- 5 -------- 6 -------- 7 Agree

3. The performance moved me.

Disagree 1 -------- 2 -------- 3 -------- 4 -------- 5 -------- 6 -------- 7 Agree

4. I'd like to see the performance again.

Disagree 1 -------- 2 -------- 3 -------- 4 -------- 5 -------- 6 -------- 7 Agree

5. The performance held my attention.

Disagree 1 -------- 2 -------- 3 -------- 4 -------- 5 -------- 6 -------- 7 Agree

6. I feel like I could relate to the performance.

Disagree 1 -------- 2 -------- 3 -------- 4 -------- 5 -------- 6 -------- 7 Agree

7. The performance confused me.

Disagree 1 -------- 2 -------- 3 -------- 4 -------- 5 -------- 6 -------- 7 Agree

8. At any moment during the performance, I was curious what would happen next.

Disagree 1 -------- 2 -------- 3 -------- 4 -------- 5 -------- 6 -------- 7 Agree

9. During the performance, I found myself thinking about other things.

Disagree 1 -------- 2 -------- 3 -------- 4 -------- 5 -------- 6 -------- 7 Agree

10. At times I felt like time stood still.

Disagree 1 -------- 2 -------- 3 -------- 4 -------- 5 -------- 6 -------- 7 Agree

11. I noticed every moment of time passing.

Disagree 1 -------- 2 -------- 3 -------- 4 -------- 5 -------- 6 -------- 7 Agree

12. Attending the performance heightened my senses and made me acknowledge my immediate surroundings more vividly.

Disagree 1 -------- 2 -------- 3 -------- 4 -------- 5 -------- 6 -------- 7 Agree

13. I feel like I experienced the same emotions as <u>the performers</u> while watching the performance.

Disagree 1 -------- 2 -------- 3 -------- 4 -------- 5 -------- 6 -------- 7 Agree

14. I feel like I experienced the same emotions as <u>other spectators</u> while watching the performance.

Disagree 1 -------- 2 -------- 3 -------- 4 -------- 5 -------- 6 -------- 7 Agree

Please circle the appropriate number in response to the following questions.

1. To what extent did you inhabit the world of the performers, lose track of time and forget about everything else?

Not at all 1 -------- 2 -------- 3 -------- 4 -------- 5 -------- 6 -------- 7 A great deal

2. How much did the performance make you think?

Not at all 1 -------- 2 -------- 3 -------- 4 -------- 5 -------- 6 -------- 7 A great deal

3. How much were you provoked or challenged by the performance?

Not at all 1 -------- 2 -------- 3 -------- 4 -------- 5 -------- 6 -------- 7 A great deal

4. To what extent did the performance cause you to reflect on your own opinions or beliefs?

Not at all 1 -------- 2 -------- 3 -------- 4 -------- 5 -------- 6 -------- 7 A great deal

5. To what extent do you feel that you understood the performance?

Not at all 1 -------- 2 -------- 3 -------- 4 -------- 5 -------- 6 -------- 7 A great deal

6. Did you have a strong emotional response to the performance?

Not at all 1 -------- 2 -------- 3 -------- 4 -------- 5 -------- 6 -------- 7 A great deal

7. To what extent did you relate to, or feel bonded with one or both of the performers?

Not at all 1 -------- 2 -------- 3 -------- 4 -------- 5 -------- 6 -------- 7 A great deal

8. To what extent was the performance therapeutic for you in an emotional sense?

Not at all 1 -------- 2 -------- 3 -------- 4 -------- 5 -------- 6 -------- 7 A great deal

9. How much did the performance leave you feeling uplifted or inspired?

Not at all 1 -------- 2 -------- 3 -------- 4 -------- 5 -------- 6 -------- 7 A great deal

10. To what degree did you pass into a different state of consciousness for a period of time?

Not at all 1 -------- 2 -------- 3 -------- 4 -------- 5 -------- 6 -------- 7 A great deal

11. To what extent did the performance leave you feeling empowered?

Not at all 1 -------- 2 -------- 3 -------- 4 -------- 5 -------- 6 -------- 7 A great deal

12. To what extent did you feel a sense of belonging or connectedness with the rest of the audience?

Not at all 1 -------- 2 -------- 3 -------- 4 -------- 5 -------- 6 -------- 7 A great deal

13. To what extent did you feel a sense of belonging or connectedness with the performers?

Not at all   1 -------- 2 -------- 3 -------- 4 -------- 5 -------- 6 -------- 7   A great deal

14. Did the performance leave you with new insight on human relations or social issues, or a perspective that you didn't have before?

Not at all   1 -------- 2 -------- 3 -------- 4 -------- 5 -------- 6 -------- 7   A great deal

15. To what extent did the measurement devices impact your experience of the performance?

Not at all   1 -------- 2 -------- 3 -------- 4 -------- 5 -------- 6 -------- 7   A great deal

Please respond to the following questions.

How often did the performers make direct eye contact with each other?

(   ) times

What three questions did the performers repeat verbally?

1.

2.

3.

Do you know <u>either or both of the performers</u> personally?

Not at all   1 -------- 2 -------- 3 -------- 4 -------- 5 -------- 6 -------- 7   Very well

If <u>yes</u>, what is the relationship between you and <u>either or both of the performers</u>?

Do you have unanswered questions that you would like to ask the performers or creators of the work?

____ No ____Yes

Would you like to discuss the meaning or merits of the performance with others who attended?

____ No ____ Yes (casual exchange) ____ Yes (intense exchange)

In roughly 100 words, can you describe what happened in the performance? Was there a specific moment that stood out to you?
