# Supplementary material for: Delta-band audience brain synchrony tracks engagement with live and recorded dance
Source: iScience. 2025 Jul 7;28(7):112922. doi: 10.1016/j.isci.2025.112922 (PMC12432844; doi:10.1016/j.isci.2025.112922)
Supplement: Document S2. Figures S1–S10, Tables S1–S4, and Data S1/Methods S1 [file mmc2.pdf]

Supplementary Tables S1-4, Figures S1-10, and Data/Methods S1 related to Figure 7.

Table S1.

Datasets in relation to social and physical liveness, available EEG measures and results summary. Small sample size and synchronisation did not allow us to compute group-based CCA for the cinema screening. All EEG data for the studio screening was lost due to a data streaming failure.

Study	Physical Liveness	Social Liveness	Summative Engagement	EEG	Continuous Engagement	
					most	least
Live	Live	Together	high	Delta CCA	high	low
				Delta PLV	high	low
				Alpha/Delta Power	low	low
Cinema	Recorded		intermediate	Delta CCA	x	x
				Delta PLV	high	low
				Alpha/Delta Power	intermediate	intermediate
Studio				Delta CCA	x	x
				Delta PLV	x	x
				EEG Power	x	x
Lab		Alone	low	Delta CCA	intermediate	low
				Delta PLV	high	low
				Alpha/Delta Power	high	high

Figure S1.

A: Relationships between correlated component 1 (C1) and Choreographer ratings for live Performances 1, 2, and 3 (P1, P2, P3). Shaded sections indicate when Performance INS > Resting-State INS ($p_{FDR} < .05$). B: Time-series of $C1_{Delta}$ for P1, P2, P3 and Lab on the same y-axis for comparison.

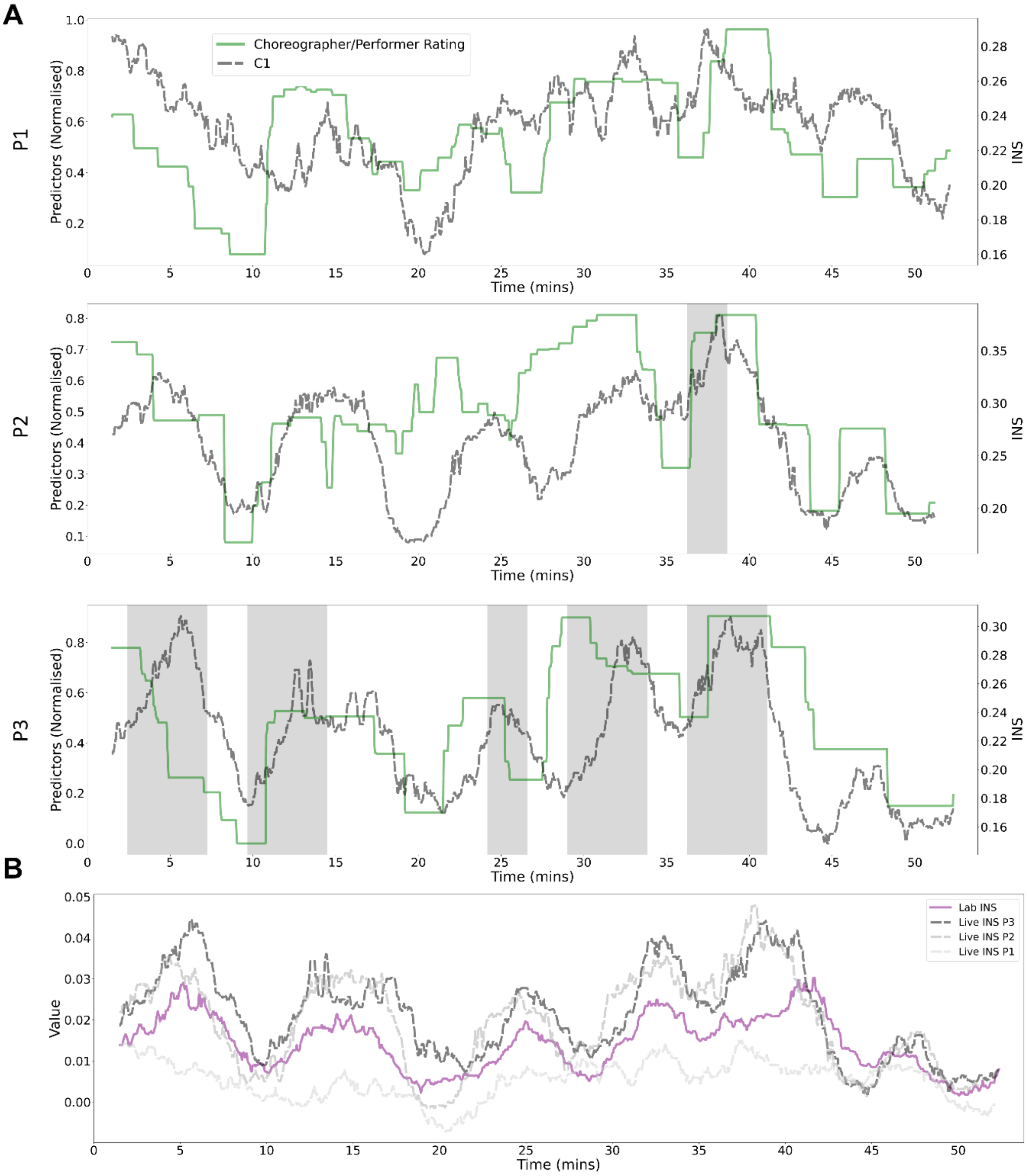

Table S2.

Summary of significant clusters for each performance and frequency band.

	Delta	Theta	Alpha	Beta
Performance 1				
No. Cluster	17	8	4	0
Cluster Size	5.06 (0.97)	3.75 (2.7)	3 (1.8)	-
Performance 2				
No. Cluster	40	29	7	2
Cluster Size	7.25 (3.2)	4.69 (3.03)	3.42 (1.7)	4 (0)
Performance 3				
No. Cluster	91	68	19	0
Cluster Size	6.60 (3.4)	4.49 (4.7)	2.53 (1.3)	-
Lab				
No. Cluster	108	89	14	2
Cluster Size	7.55 (4.3)	2.96 (3.0)	2.81 (2.5)	4 (4.2)

Table S3.

Pairwise chi-square tests comparing number of significant CCA Component 1 clusters between frequency bands for each performance.

	Delta – Theta (χ^2, p_{FDR})	Delta – Alpha (χ^2, p_{FDR})	Delta – Beta (χ^2, p_{FDR})
Performance 1	21.16, < .0001	17.19, .0001	13.24, .001
Performance 2	65.06, < .0001	43.08, < .0001	38.09, < .0001
Performance 3	155.03, < .0001	106.04, < .0001	87.04, <.0001

Table S4.

Mean percentage of outlier samples (+- 40 milliseconds) in each frequency band for each Performance, with standard deviation of the mean in brackets.

	Δ	ϑ	α	β
Performance 1	2.04 (.83)	3.15 (1.41)	5.36 (1.65)	6.67 (1.96)
Performance 2	2.19 (.84)	2.97 (1.49)	5.23 (2.0)	6.26 (2.19)
Performance 3	3.05 (1.1)	3.5 (1.51)	6.03 (2.34)	7.62 (1.94)

Figure S2.

Frequency spectrum of soundtrack audio, video luminance, and dancer accelerometer (X, Y, Z axes). The delta-band from 1 – 4 Hz is denoted by dotted vertical lines.

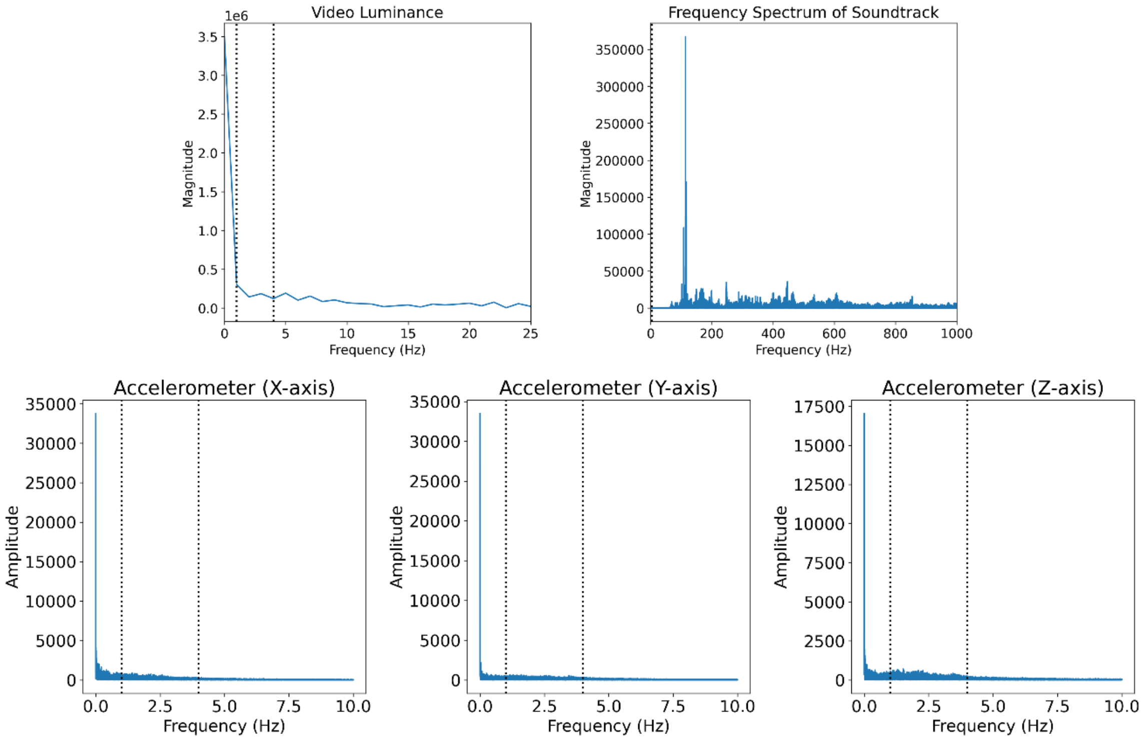

Figure S3.

Histograms depicting distributions of uncorrected p-values from pairwise Granger Causality tests for Performances 1, 2, and 3 (P1, P2, P3) with the true brain synchrony component C1, and randomised C1(Rand) as outcome variables, from lags of 1 – 15 seconds.

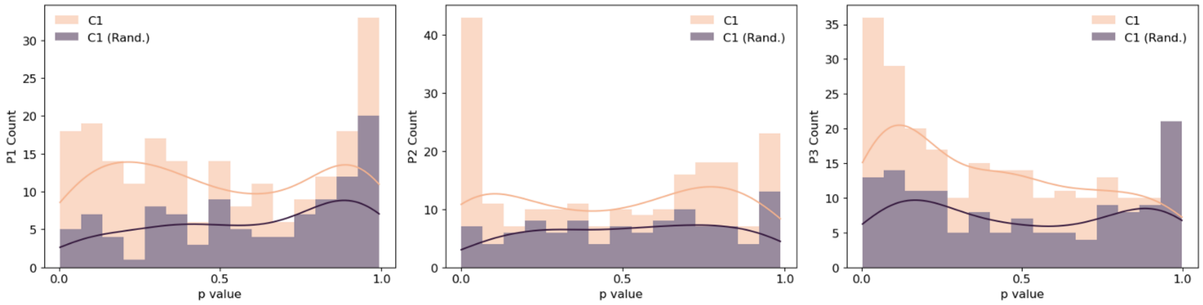

Data/Methods S1.

EEG data analyses for the cinema screening

We conducted a cinema screening study in which up to 15 co-present audience members watched an edited video recording of *Detective Work* Performance 3 together in a cinema. Due to technical errors resulting in a lack of synchronization accuracy between devices, we do not report a CCA analysis of this dataset (the INS_{Delta} component from the cinema sample explained 21% of variance, that is much lower than what we observe in the live performances 2, 3, and the lab study. However, it is similar to Performance 1, where we also found a lack of synchronization accuracy between EEG devices). The technical errors led to imprecise markers denoting the start and end of the video recording and greater variability in Lab Streaming Layer markers received by devices. Some devices did not receive LSL markers, or the photoresistor signal was too noisy to align recordings, resulting in a reduced sample size (Screening 1 n = 7; Screening 2 n = 13, Screening 3 n = 8). If EEG recordings cannot be aligned between participants to the beginning of the video recording onset, this will affect the validity of inter-brain synchrony analyses. We clarify this point further in an analysis of the LSL markers depicted in Supplementary Figure S7A and now compare these with markers from the cinema sample (Figure 7C below). A Welch's t-test showed that the difference in time between consecutive LSL markers sent every 5 seconds was greater for the live (M = 4986.6 ms, SD = 6.34) compared to the cinema sample (M = 4986.32, SD = 13.35; t = 2.85, p = .004). This finding indicates that markers sent during the live performances were more accurate than the cinema screenings.

Amplitude and phase-dependent EEG correlates of social context

CCA provides a data-driven approach to identify a genuinely group-based correlate of shared engagement that avoids making a priori and arbitrary assumptions about specific frequencies or brain topographies, but it does not reveal if higher INS results from tighter alignment of spectators' brains to the unfolding performance features (phase synchrony), heightened arousal or attentional awareness (amplitude synchrony) or a combination of both. To better understand the relative contributions of phase and amplitude synchrony to INS_{Delta},

we therefore compared Phase Locking Value (PLV) in the delta band and spectral EEG power in the alpha and delta band across live, cinema screening, and lab versions of Detective Work and explored the role of physical distance and seating position between audience members for INS.

First, we computed power spectra for live, cinema and lab performance contexts for the unison (high engagement) and pedestrian (low engagement section), see Figure S4. To specifically test if higher arousal was related to higher engagement in the lab as compared to all three live performances and the cinema experiment in alpha and delta frequency bands, we conducted a linear mixed effects model with EEG power as the outcome, and frequency band (delta, alpha), engagement (high versus low engagement sections; ‘Pedestrian’ versus ‘Unison’, as determined by the choreographer’s ratings and $C1_{delta}$), and condition (live, lab and cinema) as the predictors. The model specification was as follows: EEG power ~ 1 + frequency band + engagement + experiment + frequency:experiment + engagement:experiment + (1 | participant). There was a significant main effect for frequency (β = 0.77, t = 10.59, p < 001), experiment type comparing P1-Lab (β = -0.58, t = -2.2, p = .03), P2-Lab (β = -0.79, t = -2.91, p = .004), and Cinema-Lab (β = - 0.58, t = -2.04, p = .02), and a significant interaction between frequency and experiment (P3-Lab) (β = 0.65, t = 3.15, p = .002). The main effect of frequency showed that delta power was higher than alpha power overall, and this difference was significantly greater for P3 compared to the Lab, but there were no EEG power differences between high and low engagement sections within performances (see Figure S5, Upper row).

Second, mirroring an analysis performed by Dikker et al. (2017) we then tested if higher individual arousal was associated with greater alignment to brain synchrony of the audience as a whole. We computed correlations between individual EEG power in the alpha/delta frequency band and each spectator’s index of brain synchrony ($C1_{delta}$) relative to other spectators of the same performance or the lab experiment. Alpha/Delta power and INS were uncorrelated for all three live shows (rho = -0.01, p = .9) and the lab experiment (rho 0.17, p

= 0.4). Furthermore, no significant relationship was observed between mean delta power and $C1_{delta}$ for the live (rho = 0.12, p = .39) and lab (rho -0.29, p = 0.13) experiments (see Figure S6 for scatterplots of these relationships). Greater similarity of individual spectators' delta band activity to the group was thus unrelated to their arousal during the performance.

Finally, as a purely phase dependent measures of INS we computed Phase Locking Value (PLV) between all possible pairs of audience members within each performance, the cinema screenings, and the lab study. As $C1_{Delta}$ was characterised by correlations in the delta band across occipital and central regions (see topoplots in Figs. 3 & 6), we chose to compute synchrony across these electrode positions (C3, C4, Cz, O1, Oz, O2) and for the delta band only. A linear mixed effects model was conducted with PLV_{Delta} as the outcome variable, and engagement (high versus low), performance context (live, cinema and lab), region (occipital versus central) and their respective interactions as fixed factors, while accounting for random variability by unique participant pair. The model specification was as follows: PLV ~ 1 + engagement + frequency + region + condition + engagement:condition + engagement:region (1 | pair ID).

There was a significant main effect of engagement (β = 0.03, t = 12.41, *p* < .001), such that pairwise synchrony in all cases was higher during high engagement compared to low engagement sections (see Fig S5 below). There was a significant interaction between engagement and region (β = .012, t = 2.43, *p* = .02), with higher PLV_{Delta} in occipital compared to frontal regions during high engagement sections. There was a significant interaction between engagement and condition for Lab versus P3 (β = .01, t = 2.23, *p* = .03), Lab versus Cinema (β =-.03, t = 4.55, *p* < 001), and marginally for Lab versus P2 (β = .01, t = 1.96, *p* = .05), with higher PLV synchrony for the live and cinema screening audiences during high engagement sections compared to the Lab, and vice versa for low engagement sections (see Figure S5). Post-hoc tests showed that during high engagement sections, the cinema was significantly higher than P1 (*p* = .01) and the Lab (*p* = .004). The intra class coefficient for random effects suggested a small influence of participant pair (max. ICC = 0.027).

To test for the influence of audience seating position on pairwise synchrony, pairwise distances between audience seats for the live performances were entered into an LMM as a covariate, with PLV_{Delta} as the outcome and engagement as a fixed factor. However, this did not produce a significant main effect (p = .92; see Figure S5). Further, there was no significant interaction effect between distance and engagement (p = .7). This finding suggests that shared low-level sensory features such as visual perspective do not predict brain synchrony.

Together, these results demonstrate that Delta PLV and EEG power primarily reflect differences between collective and individual viewing conditions. Moreover, PLV_{Delta} as an amplitude- independent measure of brain synchrony is higher during high than low engagement sections. Furthermore, choreographer engagement ratings Granger-cause INS_{Delta}, unlike mean audience EEG power. The possibility that visual perspective or other low-level performance features drive Delta-INS is not supported as no relationship was found between pairwise seating distance and PLV_{Delta}. While it is possible that differences in arousal between live and lab conditions did influence INS_{Delta}, EEG power did not correlate with INS, and did vary together with high and low $C1_{Delta}$ sections within the performance.

The finding that pairwise synchrony is higher in the cinema than during live performances and the lab study support our hypothesis that scene changes in the edited video recording increase INS (e.g., Hollywood video clips rich with scene cuts elicit stronger inter-subject correlations than educational videos; Petroni et al., 2018). The main effect of engagement level is still maintained even in the Cinema sample, with higher PLV_{Delta} for high compared to low engagement sections. The proportion of close-up versus wide cinematic shots in the Pedestrian versus Unison sections did not differ significantly ($\chi^2 = 3.28$, p = .07), so this difference cannot be explained by video editing.

Figure S4.

Power spectrum for live, cinema, and lab studies. Highlighted sections indicate significant difference between high (Unison) versus low (Pedestrian) engagement sections based on a signed ranks test.

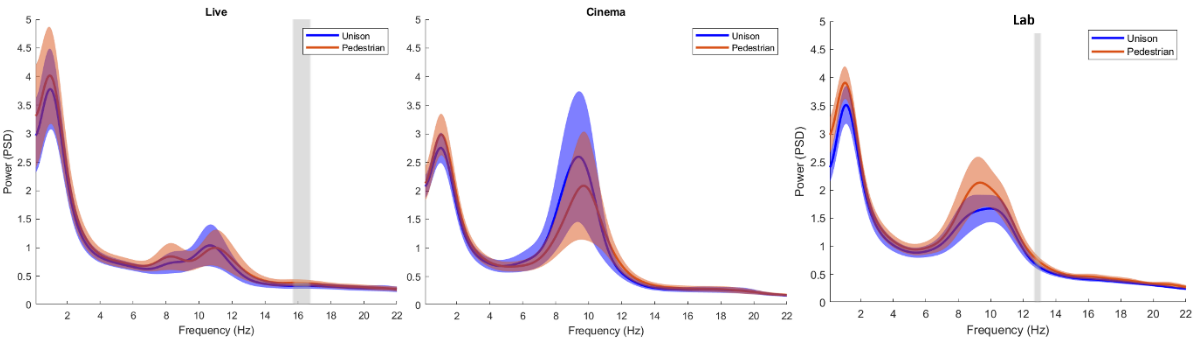

Figure S5.

Upper row: Mean EEG power for the live (P1, P2, P3, cinema screening, and lab samples in the alpha (left) and delta (right) frequency band. Lower row left: Mean pairwise synchrony (Phase Locking Value; PLV_{Delta}) for all possible pairs of participants in the live, screening, and lab studies. Lower row right: Scatterplot of non-signifciant relationship between PLV_{Delta} and pairwise distances in seating position for the live studies.

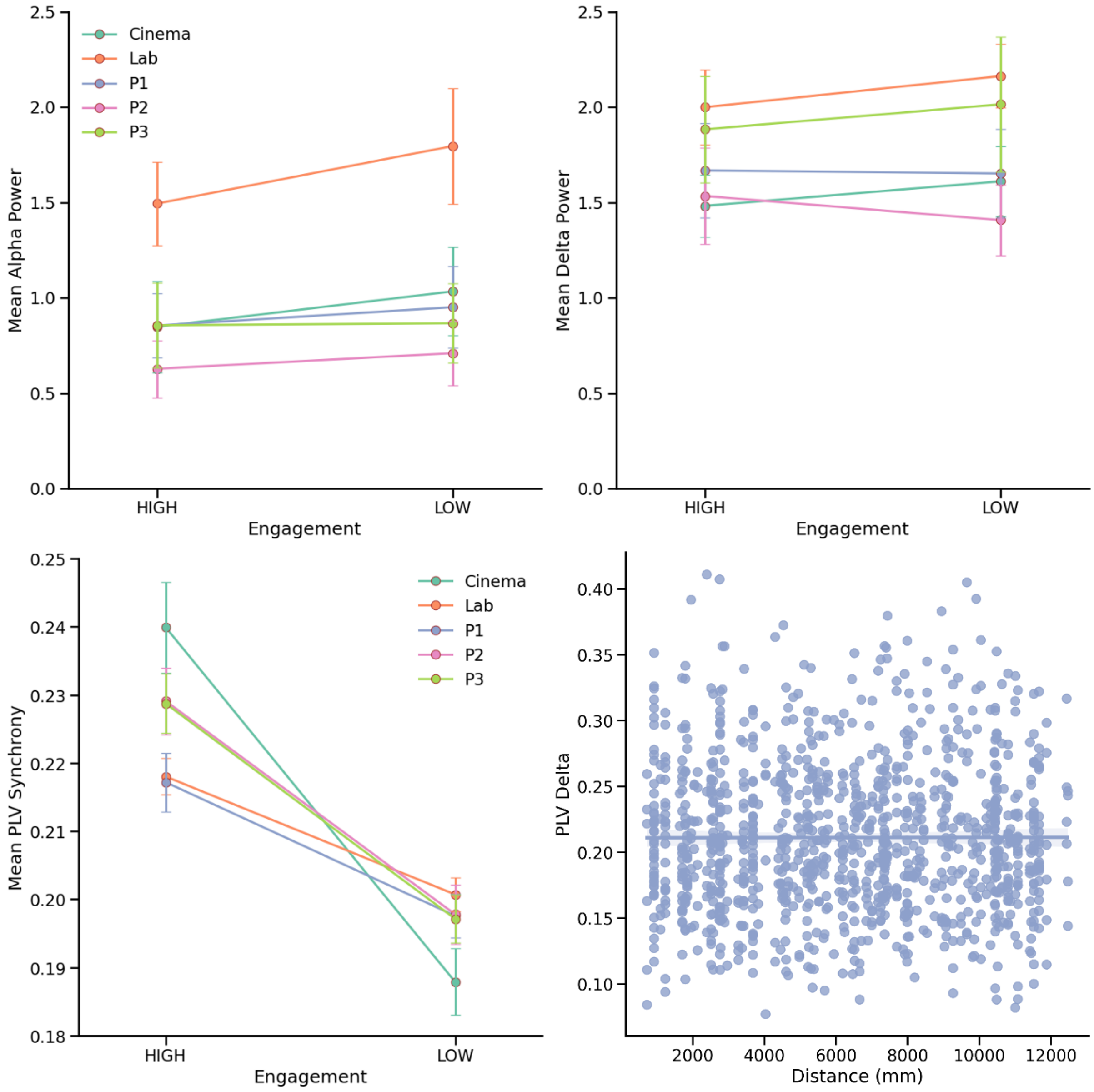

Figure S6.

Scatterplots depicting relationships between alpha, delta, EEG power, and C1$_{Delta}$ for each of the three live performances (P1, P2, P3) and the lab experiment.

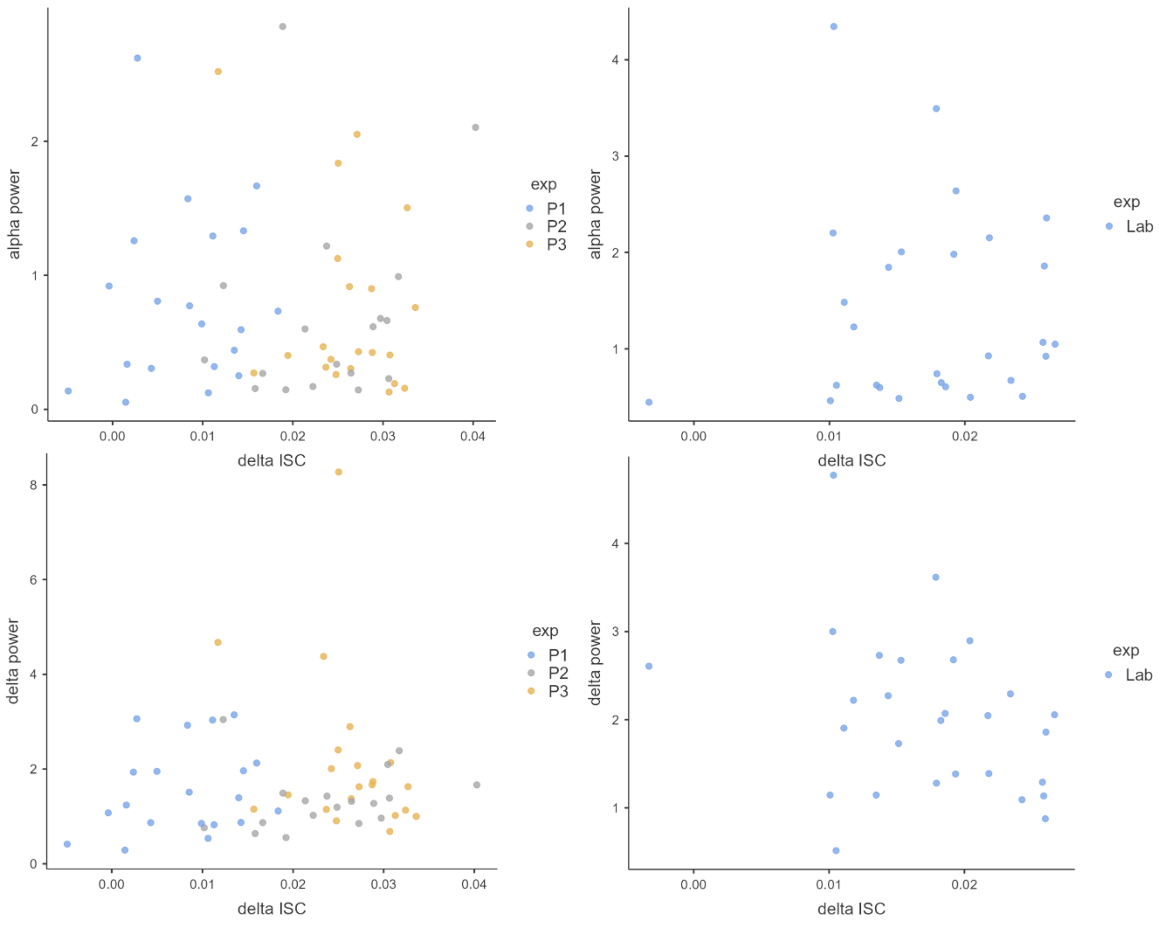

Figure. S7.

Panel A: Lab Streaming Layer timing differences between consecutive markers sent from a remote computer every 5-seconds for each participant in P1, P2, P3.

Panel B: Photoresistor timing differences between lighting change points for each participant in P1, P2, P3. Reference channel point marked in green. Mean offset from reference channel. Greater variability of LSL markers in Performance 1 likely resulted from some devices erroneously streaming EEG data to the local network whilst also being recorded locally on the device. For P2 and P3 all devices were set to only record locally on devices.

C: Lab Streaming Layer timing differences between consecutive markers sent from a remote computer every 5 seconds for each participant in Cinema Screenings 1-3.

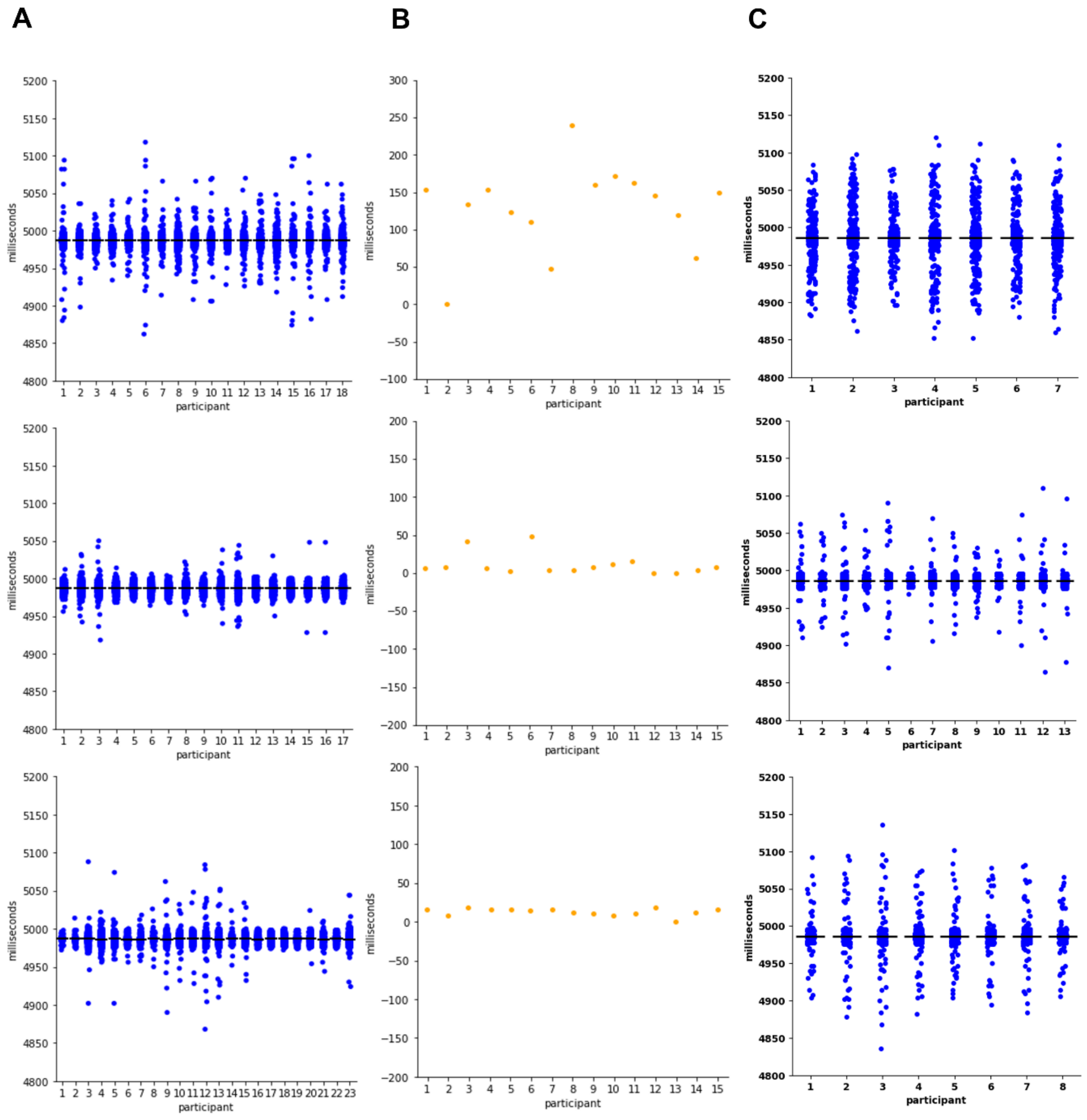

Figure S8.

Image of EEG set-up procedure in research room before the live performances. Photo Credit: Hugo Glendinning. Images of the Studio and Cinema screenings.

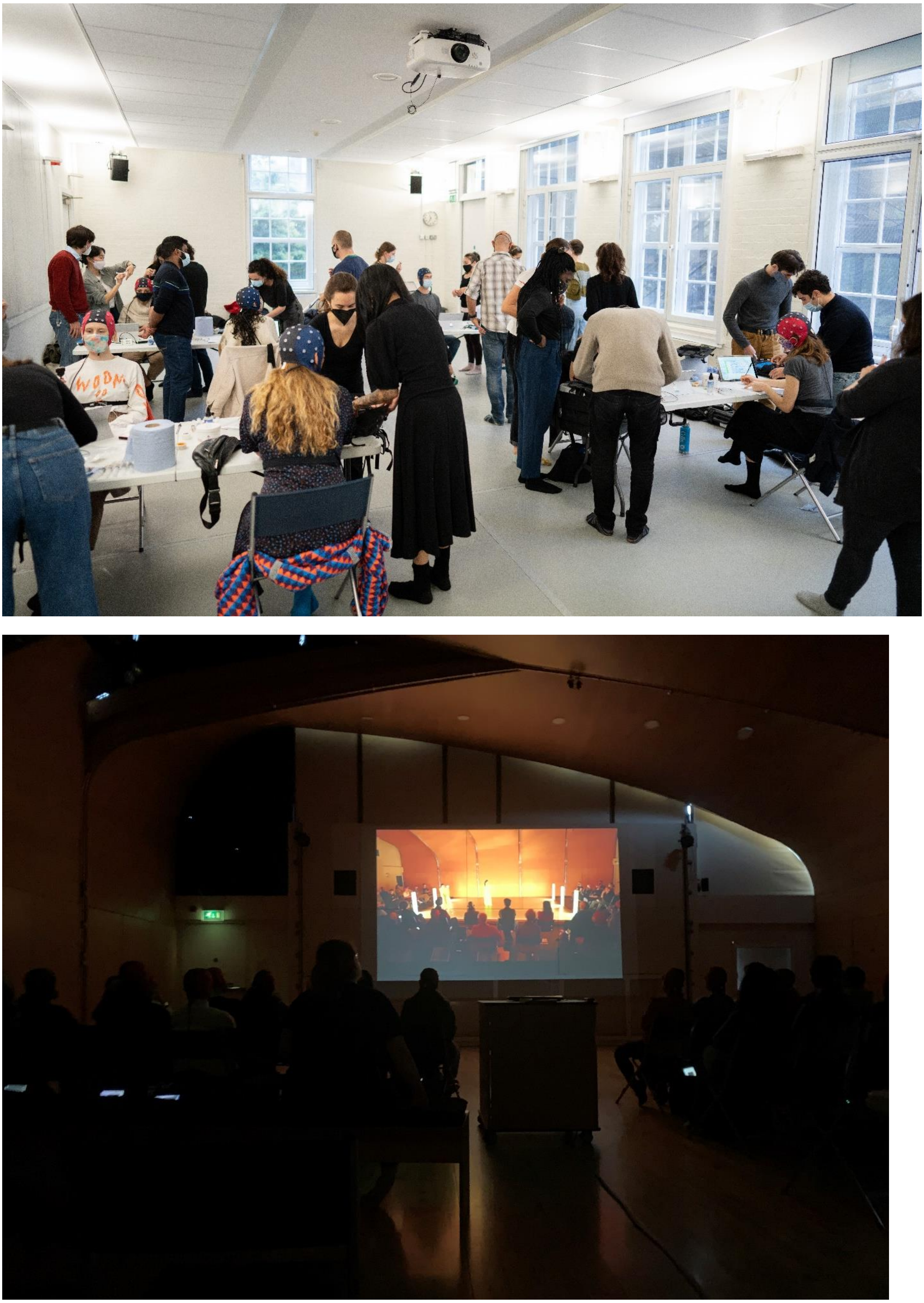

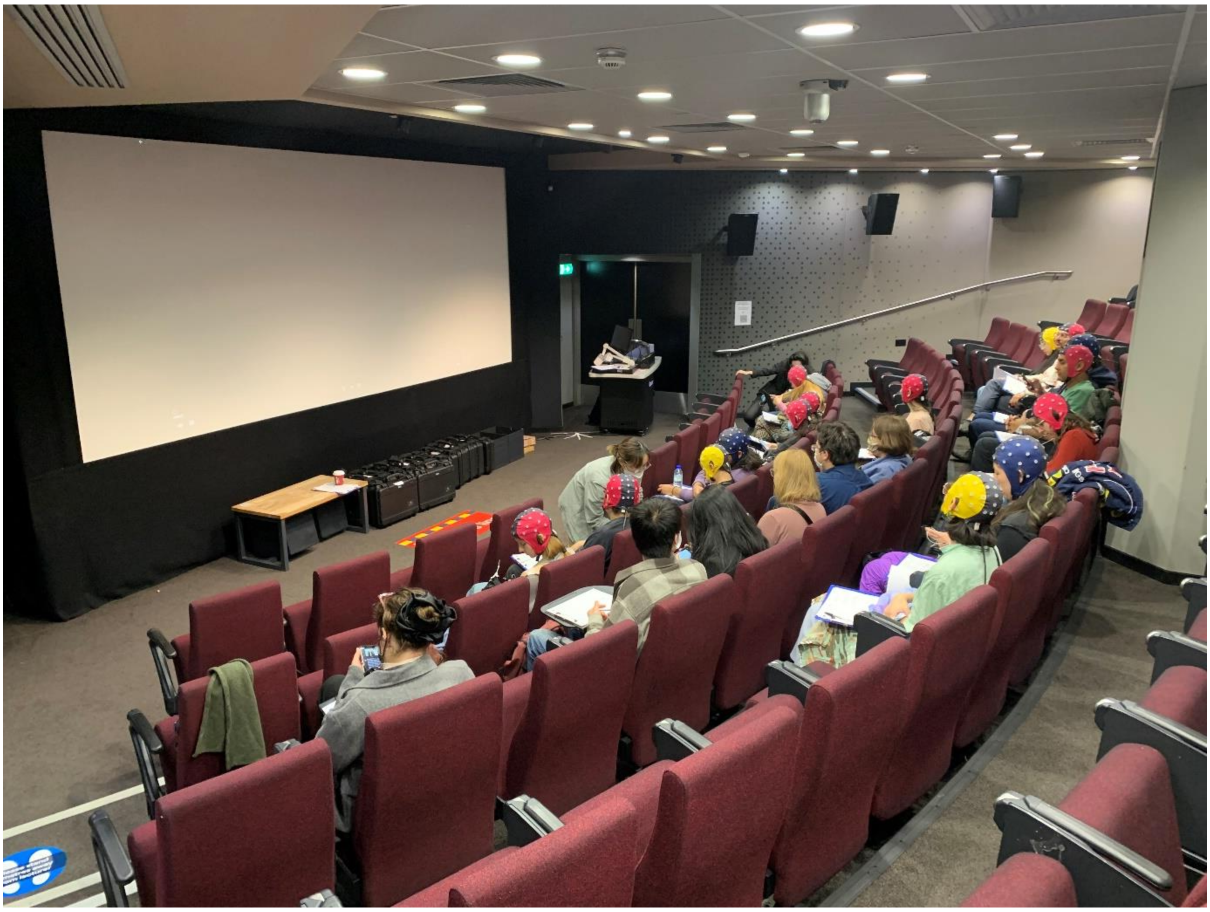

Figure S9.

Movement tracking of dancer's movements on stage using position coordinates from video camera positioned above the stage. Variability in movements is observed between the three performances.

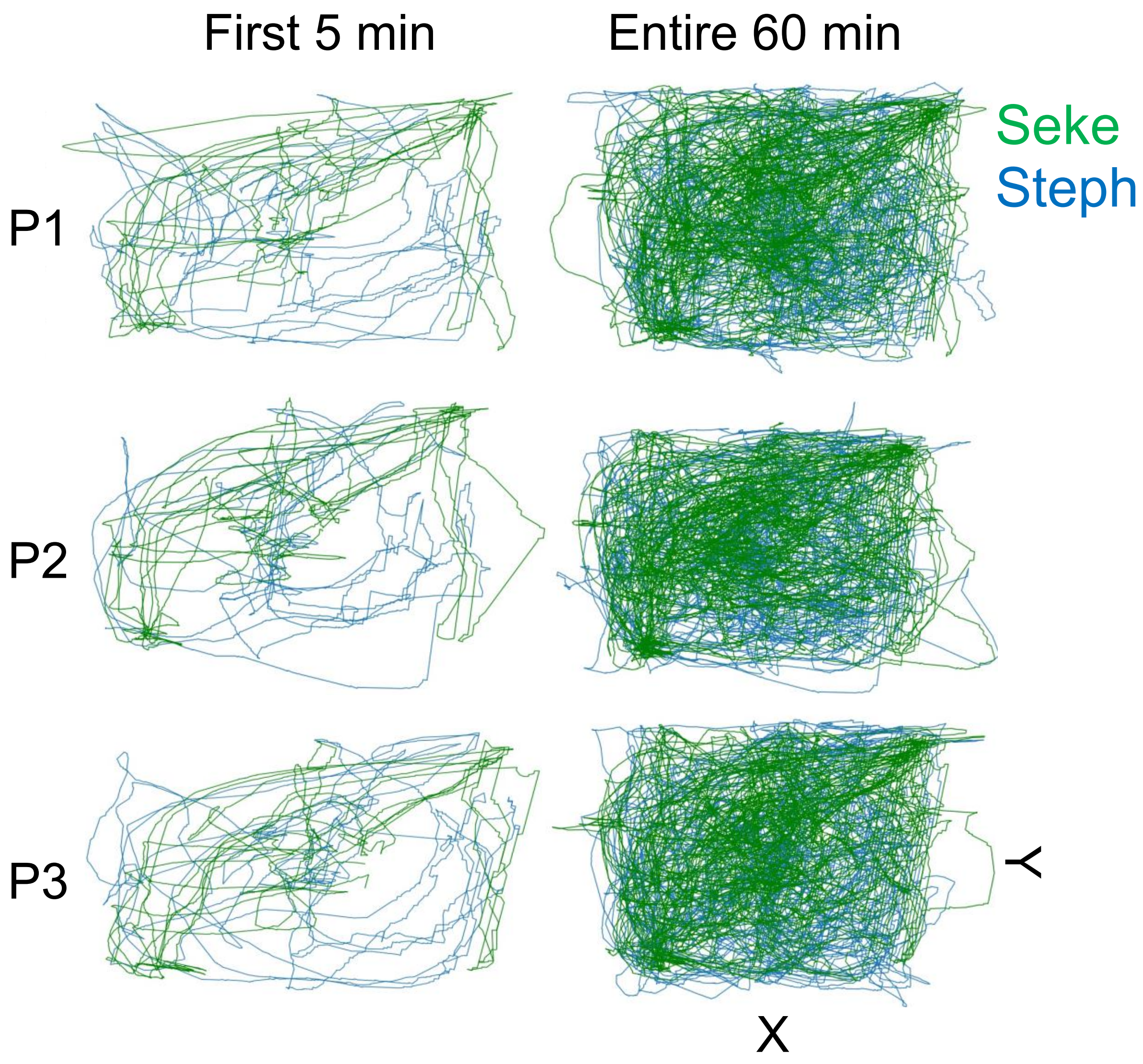

Figure S10.

A: Data from Performance 1 showing stages of synchronisation. Row 1: Photoresistor signals for each EEG device, Row 2: Reference light sensor from Mbient sensor placed close to stage, Row 3: Corresponding acceleration data from the same reference sensor, Row 4: Mbient sensor acceleration signals for wrists of each performer. Synchronisation is achieved using a combination of blackouts (between light sensors) and sync movements (between Mbient sensors). B: Light signals for Perf. 1: Change points circled at the onset of initial blackout pre- and post- shift relative to the Reference channel.

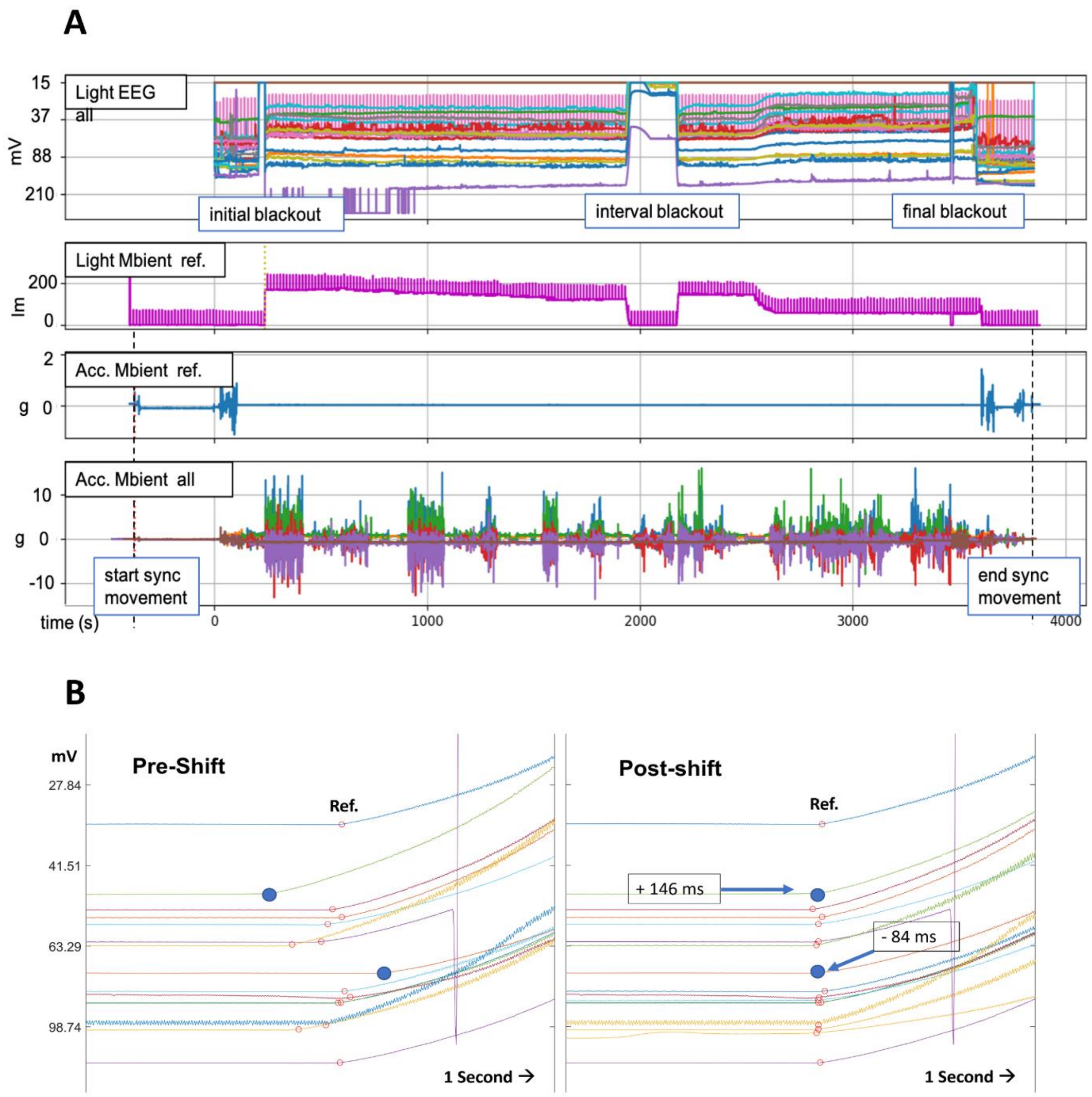

Supplementary References

Dikker, S., Wan, L., Davidesco, I., Kaggen, L., Oostrik, M., McClintock, J., Rowland, J., Michalareas, G., Van Bavel, J. J., Ding, M., & Poeppel, D. (2017). Brain-to-Brain Synchrony Tracks Real-World Dynamic Group Interactions in the Classroom. *Current Biology*, *27*(9), 1375–1380. https://doi.org/10.1016/j.cub.2017.04.002

Petroni, A., Cohen, S. S., Ai, L., Langer, N., Henin, S., Vanderwal, T., Milham, M. P., & Parra, L. C. (2018). The Variability of Neural Responses to Naturalistic Videos Change with Age and Sex. *eNeuro*, *5*(1). https://doi.org/10.1523/ENEURO.0244-17.2017
